# Estrogen receptor α and aryl hydrocarbon receptor independent growth inhibitory effects of aminoflavone in breast cancer cells

**DOI:** 10.1186/1471-2407-14-344

**Published:** 2014-05-20

**Authors:** Ashley M Brinkman, Jiacai Wu, Karen Ersland, Wei Xu

**Affiliations:** 1 Molecular and Environmental Toxicology Center, University of Wisconsin – Madison, Madison, WI, USA; 2 Department of Oncology, McArdle Laboratory for Cancer Research, University of Wisconsin – Madison, Madison, WI, USA; 3 Center for Science Research, Guilin Medical University, Guilin, Guangxi, China; 4 University of Wisconsin Carbone Cancer Center Flow Cytometry Laboratory, University of Wisconsin – Madison, Madison, WI, USA

**Keywords:** Aminoflavone, Breast cancer, Estrogen receptor, Aryl hydrocarbon receptor, Knockdown cell lines

## Abstract

**Background:**

Numerous studies have implicated the aryl hydrocarbon receptor (AhR) as a potential therapeutic target for several human diseases, including estrogen receptor alpha (ERα) positive breast cancer. Aminoflavone (AF), an activator of AhR signaling, is currently undergoing clinical evaluation for the treatment of solid tumors. Of particular interest is the potential treatment of triple negative breast cancers (TNBC), which are typically more aggressive and characterized by poorer outcomes. Here, we examined AF’s effects on two TNBC cell lines and the role of AhR signaling in AF sensitivity in these model cell lines.

**Methods:**

AF sensitivity in MDA-MB-468 and Cal51 was examined using cell counting assays to determine growth inhibition (GI_50_) values. Luciferase assays and qPCR of AhR target genes cytochrome P450 (*CYP*) *1A1* and *1B1* were used to confirm AF-mediated AhR signaling. The requirement of endogenous levels of AhR and AhR signaling for AF sensitivity was examined in MDA-MB-468 and Cal51 cells stably harboring inducible shRNA for AhR. The mechanism of AF-mediated growth inhibition was explored using flow cytometry for markers of DNA damage and apoptosis, cell cycle analysis, and β-galactosidase staining for senescence. Luciferase data was analyzed using Student’s *T* test. Three-parameter nonlinear regression was performed for cell counting assays.

**Results:**

Here, we report that ERα-negative TNBC cell lines MDA-MB-468 and Cal51 are sensitive to AF. Further, we presented evidence suggesting that neither endogenous AhR expression levels nor downstream induction of AhR target genes *CYP1A1* and *CYP1B1* is required for AF-mediated growth inhibition in these cells. Between these two ERα negative cell lines, we showed that the mechanism of AF action differs slightly. Low dose AF mediated DNA damage, S-phase arrest and apoptosis in MDA-MB-468 cells, while it resulted in DNA damage, S-phase arrest and cellular senescence in Cal51 cells.

**Conclusions:**

Overall, this work provides evidence against the simplified view of AF sensitivity, and suggests that AF could mediate growth inhibitory effects in ERα-positive and negative breast cancer cells, as well as cells with impaired AhR expression and signaling. While AF could have therapeutic effects on broader subtypes of breast cancer, the mechanism of cytotoxicity is complex, and likely, cell line- and tumor-specific.

## Background

Aside from non-melanoma skin cancers, breast cancer is the most common cancer among women worldwide, with nearly 1.4 million new cases diagnosed in 2008 [1]. Often, breast cancers are characterized by their expression of hormone receptors (estrogen receptor, ER; progesterone receptor, PR; or human epidermal growth factor receptor 2, HER2). Cancers expressing one or more of these receptors have the potential to be treated with targeted therapies, including tamoxifen and trastuzumab. On the other hand, there is no specific treatment regimen for patients whose cancers lack these three receptors, so called triple-negative breast cancers (TNBC), which tend to be clinically aggressive with a trend of poorer outcomes [2]. Thus, it is critical to develop and explore therapeutic options that may be of use to these patients.

Aminoflavone (AF; 4H-1-benzopyran-4-one, 5-amino-2-(4-amino-3-fluorophenyl)-6,8-difluoro-7-methyl, NSC 686288) is a synthetic flavonoid compound [3]. Similar compounds are frequently found in fruits and vegetables, and have a variety of effects within the body, including reported cytostatic, apoptotic, anti-inflammatory, anti-angiogenic, and estrogenic activities [4]. The National Cancer Institute’s 60 human tumor cell line anticancer drug screen revealed that AF mediated growth inhibition in numerous renal, breast and ovarian tumor cell lines, and produced a unique “fingerprint” of activity in the COMPARE algorithm, unlike any other group of anti-tumor compounds [5-7]. A pattern uncovered in AF’s differential activity in human breast cancer cell lines was the exquisite sensitivity of cells expressing estrogen receptor alpha (ERα), such as MCF7 and T47D, and resistance exhibited by cells lacking ERα expression, including MDA-MB-231, Hs578T, and BT-549. When mice bearing ERα-positive MCF7 xenografts were treated with AF, tumor growth was inhibited [8]. Further, it has been shown that AF-resistant and ERα negative cell lines MDA-MB-231 and Hs578T may be re-sensitized to AF through co-treatment with vorinostat, which reactivates ERα expression and AhR-mediated CYP1A1 activity [9]. These data imply that ERα-positive cancers might exhibit enhanced sensitivity to AF as compared with ERα-negative cancers.

Before the cytotoxic mechanism of AF was studied in ERα-positive breast cancer cell lines, other flavonoid analogs had been synthesized/extracted and examined [10-12]. Growth inhibition exerted by these related compounds is attributed to a number of processes, including topisomerase inhibition, blocking of tubulin polymerization, and decreases in protein kinase activity [13-15]. However, AF’s COMPARE fingerprint differs from compounds with these mechanisms of action, suggesting that the antiproliferative activity of AF is the result of a different mechanism [7,8]. Because flavonoid compounds have been shown to bind the intracellular aryl hydrocarbon receptor (AhR) and activate the AhR signaling pathway, one suggestion to explain AF’s activity pattern is metabolic activation by the AhR and its target genes, specifically the 1A isoforms of cytochome P450 (CYP) enzymes [7,8,16,17]. An AhR-deficient clone of MCF7 that was generated by continuous exposure to 100nM benzo [a] pyrene for six to nine months (Ah^R100^) has been shown to be rendered resistant to AF [8,18,19]. Further, previous studies revealed that AF is metabolized by CYP1A1 and, to a lesser extent, 1A2 and 1B1, and that this metabolism produces hydroxylamine species [7,8,17]. It has also been shown that AF induces expression of sulfotransferase (SULT) 1A1 enzymes in AF-sensitive MCF7 cells, and that transfection of SULT1A1 into resistant MDA-MB-231 cells restores sensitivity [20]. Correlations between high activity *CYP1A1* and *SULT1A1* alleles and sensitivity to AF have also been made in chinese hampster cells engineered to express various polymorphisms of these genes [21]. AF metabolites, presumably though the CYP/SULT driven bioactivation pathway, have been shown to be DNA damaging agents, inducing DNA-protein crosslinks, cytokeratin-RNA crosslinks, phosphorylation of p53,increased expression of p21, γ-Histone 2AX (γ-H2AX), reactive oxygen species-mediated apoptosis, and S-phase arrest in sensitive populations of cells [7,8,17,19,20,22-25]. These studies implicated that AhR might, at least in part, mediate the cytotoxic and DNA damaging effects of AF.

AhR is a ligand-activated transcription factor that is known for its role in mediating the cellular response to dioxins, polycyclic aromatic hydrocarbons, and related compounds [26,27]. Upon ligand binding, conformational changes occur, allowing AhR’s nuclear localization signal to be exposed. This leads to translocation of AhR to the nucleus, where AhR dimerizes with aryl hydrocarbon receptor nuclear translocator (ARNT), and binds to dioxin responsive elements (DREs), resulting in regulation of target genes [28,29]. Of particular importance regarding the bioactivation of AF are AhR target genes in the *CYP1A* family [7,8,17]. In addition to increasing *CYP1A1/1A2/1B1* expression, AF induces nuclear translocation of AhR and stimulates protein-DNA complexes formed on DREs in AF-sensitive MCF7 human breast cancer cells, suggesting that AF is an AhR agonist [8]. Further, localization of AhR in the cellular cytoplasm has been shown to correlate with AF sensitivity [8,19]. Interestingly, it has also been shown that AF inhibits hypoxia inducible factor 1α (HIF1α), a protein which may interact with AhR [30]. However, it remains to be determined whether AhR expression and downstream gene activation serve as determinants for AF sensitivity, particularly in ERα-negative human cell lines.

The objective of this study was to further investigate potential biomarkers of AF sensitivity, including ERα expression, AhR expression, and AhR signaling in human breast cancer cell lines. Here, we demonstrate that two ERα-negative human breast cancer cell lines, MDA-MB-468 and Cal51, exhibit sensitivity to AF, and the sensitivity is retained after knockdown of AhR protein [23] . While both cell lines express high levels of endogenous AhR protein, they display differential abilities to induce AhR target genes *CYP1A1* and *CYP1B1*, yet the cytotoxicity of AF in these cell lines remains similar. To our knowledge, and using the cBio portal maintained by the Computational Biology Center at Memorial Sloan-Kettering Cancer Center, neither of these human breast cancer cell lines harbors a mutation in the AhR gene. These results suggest that neither expression of ERα and AhR nor *CYP* induction is necessarily predictive of AF sensitivity. Further, we showed that AF exerts its antiproliferative activity in a cell-type specific manner: low dose AF treatment causes DNA damage, S-phase arrest and apoptosis in MDA-MB-468 AhR knockdown cells (MDA-MB-468shAhR), while causing DNA damage, S-phase arrest, and a senescent-like phenotype in Cal51 AhR knockdown cells (Cal51shAhR).

## Methods

### Chemicals

Doxycycline (Dox) was obtained from Clontech (Mountain View, CA). β-Naphthoflavone (BNF) was obtained from Sigma (St. Louis, MO). Aminoflavone (AF) was obtained from the Developmental Therapeutics Program Repository of the National Cancer Institute at Frederick (Frederick, MD). BNF and AF were stored in dimethyl sulfoxide (DMSO). Triton X-100 was obtained from Fisher (Fair Lawn, NJ), protease inhibitors were obtained from Roche Scientific (Basel, Switzerland), and benzonase was obtained from Novagen (San Diego, CA). All other chemicals were obtained from Sigma (St. Louis, MO).

### Cell culture

Cell culture media were obtained from Invitrogen (Carlsbad, CA). MDA-MB-468, Cal51, 293 T, and 101 L hepatoma cells were maintained in Dulbecco’s Modified Eagle’s Medium (DMEM) with 10% Gibco Fetal Bovine Serum (FBS, Invitrogen) at 37°C and 5% CO_2_. MDA-MB-468shAhR and Cal51shAhR were maintained in DMEM with 10% Tet-System Approved FBS (Clontech) at 37°C and 5% CO_2_. MDA-MB-468 cells are mammary adenocarcinoma cells from a pleural effusion and were purchased from ATCC (Manassas, VA). Cal51 cells are also mammary adenocarcinoma cells from a plural effusion, but they exhibit a normal karyotype [31]. Cal51 was purchased from DSMZ (Braunschweig, Germany). 101 L hepatoma cells harbor a stably transfected luciferase reporter driven by three upstream DREs, and were obtained from Dr. Christopher Bradfield (Madison, WI), initially acquired from the laboratory of Dr. Robert Tukey (San Diego, CA) [32]. Parental cell lines were maintained in our laboratory for less than six months after resuscitation.

### Dioxin responsive element reporter assays

101 L cells were seeded in triplicate at 2.2 × 10^4^ cells/well on a clear 48-well tissue culture plate in phenol red-free DMEM with 5% charcoal-stripped FBS. After 24 hours, media were removed and replaced with media containing 0.1% dimethyl sulfoxide (DMSO) or a range of AF doses (100nM, 500nM, 1 μM, 10 μM). After 18 hours of compound treatment, the cells were washed with 50 μL 1× PBS (Gibco, Invitrogen) and lysed with 50 μL Tropix lysis buffer (100 mM K_2_HPO_4_, 0.2% Triton X-100, pH 7.8, Applied Biosystems). Cell lysate was mixed 1:1 with luciferase substrate (Promega, Madison, WI), and luminescence was measured with a 700-nm filter on a Victor X5 microplate reader (PerkinElmer, Waltham, MA). The Bradford method (Bio-Rad) was used to measure total protein in each sample. Raw luciferase data was normalized to both total protein and background luciferase expression in the DMSO control samples and expressed as fold-increase over DMSO.

### Inducible knockdown of AhR by lentiviral infection

pSUPER vectors were constructed using two previously published siRNA sequences directed toward the AhR, 5′CAGACAGUAGUCUGUUAUA 3′ and 5′CGUUUACCUUCAAACUUUA 3′, by standard cloning procedures [33-35]. The siRNA cassette downstream of the H1 promoter was sequenced to confirm accuracy (University of Wisconsin Biotechnology Center, Madison, WI), excised from pSUPER, and subcloned into the lentiviral vector pLVTHM. Viral particles containing shAhR vectors were created by transfecting host 293 T cells with vectors encoding for VSVG, a lentiviral vector coat protein, PAX2, a packaging plasmid, and pLVTHM-shAhR using standard protocols [36]. Briefly, subconfluent 293 T cells were transfected with 0.5 μg VSVG, 1 μg PAX2, and 1.5 μg pLVTHM-shAhR using Trans-IT LT1 transfection reagent (Mirus Bio, Madison, WI). After six hours, medium was changed and recombinant lentivirus vectors were harvested 24 hours later. Using a similar protocol, pLV-tTR-KRAB recombinant lentivirus was produced. pLV-tTR-KRAB encodes a tetracycline (Tet)- controlled hybrid protein containing the Tet repressor (tTR) and the Krüppel associated box (KRAB) domain of human Kox1 [37,38]. The purpose of KRAB in Tet-responsive systems is described elsewhere (34). MDA-MB-468 and Cal51 cells were seeded subconfluently in a six-well tissue culture plate at 37°C and 5% CO_2_. Twenty-four hours later, media were removed and replaced with 1 mL of DMEM supplemented with 10% FBS containing recombinant pLV-tTR-KRAB and 5 μg/mL polybrene. After allowing two passages for recovery, the MDA-MB-468 and Cal51 cells were subjected to the same protocol, substituting pLV-tTR-KRAB with the two pLVTHM-shAhR lentiviruses, producing MDA-MB-468shAhR and Cal51shAhR cell lines.

### Western blot analysis

MDA-MD-468shAhR and Cal51shAhR were treated for seven days with vehicle or 750 ng/mL doxycycline (Dox) in DMEM with 10% Tet-Approved FBS. After treatment, cells were collected by trypsinization, washed with 1× PBS (Gibco, Invitrogen), and lysed using Triton X-100 lysis buffer (50 mM Tris pH 8.0, 400 mM NaCl, 10% glycerol, 0.5% triton X-100, protease inhibitors, and benzonase). Total protein concentration was measured using the Bradford method (BioRad), and 20 μg of protein was resolved using SDS-PAGE on 8% polyacrylamide gels. Protein was transferred to a nitrocellulose membrane at 4°C for one hour at 0.35A. Membranes were blocked with 5% nonfat milk in PBS + 0.1% Tween for one hour at room temperature, then incubated with 1:10,000 anti-AhR antibody (Santa Cruz, sc-5579) or 1:10,000 anti-β-Actin (Sigma, A5316) overnight at 4°C. Membranes were incubated with 1:10,000 goat anti-rabbit HRP or anti-mouse HRP secondary antibody for one hour at room temperature. Enhanced chemiluminescence reagents (Thermo Scientific) were applied to the membranes prior to exposure to x-ray film (Kodak).

### Cell counting assays

MDA-MB-468, MDA-MB-468shAhR, MCF7, MDA-MB-231, Cal51, and Cal51shAhR were seeded at 20,000 cells/well (MDA-MB-468, MDA-MB-468shAhR, MDA-MB-231) and 15,000 cells/well (MCF7, Cal51, Cal51shAhR), each in triplicate 12-well tissue culture plates in DMEM + 10% FBS at 37°C and 5% CO_2_. AhR knockdown cells were pretreated with vehicle or 750 ng/mL Dox for seven days prior to seeding in 12-well tissue culture plates to achieve knockdown of AhR. During AF treatment, vehicle/Dox treatments were continued. All cell lines tested were treated with AF for seven days prior to analysis. Approximate GI_50_ value, which is the concentration of compound that inhibits cell growth by 50% compared to control, was calculated using GraphPad Prism Software (Version 5.04; Graph-Pad Software Inc., San Diego, CA) and a three-parameter log versus inhibition nonlinear regression. GI_50_ values are expressed as the 95% confidence interval.

### Gene expression analysis

MDA-MB-468, MDA-MB-468shAhR, Cal51, and Cal51shAhR cells were cultured in phenol red-free DMEM + 10% charcoal stripped FBS at 37°C and 5% CO_2_ for three days prior to experiment to remove residual estrogens. Triplicate 80% confluent six cm tissue culture dishes of MDA-MB-468 and Cal51 were treated with 0.1% DMSO, 1 μM AF, or 1 μM BNF for six hours. MDA-MB-468shAhR and Cal51shAhR were pretreated with vehicle or 750 ng/mL Dox for seven days prior to seeding onto triplicate six cm tissue culture dishes, and then treated with 0.1% DMSO, 1 μM AF, or 1 μM BNF for six hours in the presence or absence of 750 ng/mL Dox. Total RNA was extracted using HP Total RNA Kit (VWR Scientific, West Chester, PA) according to the manufacturer’s protocol. Two micrograms of RNA were reverse transcribed using Superscript II RT according to the manufacturer’s protocol (Invitrogen). Fast Start Universal SYBR Green Master Mix (Roche) was used to perform qPCR for *CYP1A1* on a BioRad CFX-96 instrument, using *RPL13A* as a housekeeping gene (BioRad). The primer sequences are as follows: *CYP1A1* For 5′TGCAGA AGATGGTCAAGGAG 3′*, CYP1A1* Rev 5′ AGCTCCAAGAGGTCCAAGA 3′. *CYP1B1* For 5′CTGGATTTGGAGAACGTACCG 3′, *CYP1B1* Rev 5′TGATCCAATTCTGCCTGCAC 3′. *SULT1A1* For 5′GGCCTGATGACCTGCTCATC 3′. *SULT1A1* Rev 5′TCATGTCCAGAATCTGGCTTACC 3′. *RPL13A* For 5′ CATCGTGGCTAAACAGGTACT G 3′, *RPL13A* Rev 5′ GCACGACCTTGAGGGCAGCC 3′.

### Propidum iodide staining

AF’s ability to alter the cell cycle in MDA-MB-468shAhR and Cal51shAhR cells was analyzed using a propidium iodide (PI) staining assay according to manufacturer’s protocols (Sigma). Briefly, MDA-MB-468shAhR cells were seeded into six-well tissue culture plates and treated with 0.1% DMSO or 25nM AF for 4, 24, 48, 72, or 120 hours. Cal51shAhR cells were seeded into six-well tissue culture plates and treated with 0.1% DMSO or 250nM AF for 24, 48, 72, 120, or 168 hours. Triplicate samples were collected for all controls, and duplicate samples were collected for all treatment groups. Cells were harvested and fixed with EtOH up to a concentration of 70%, and kept at 4°C until PI staining. Samples were then analyzed by a FACScalibur instrument (Becton Dickinson) for cell cycle alterations. Data was analyzed using ModFitLT 3.2.1.

### Analysis of apoptosis and DNA damage

AF’s ability to induce apoptosis and DNA damage in MDA-MB-468 and Cal51 cells was analyzed using an Apoptosis, DNA Damage, and Cell Proliferation flow cytometry kit (BD, #562253), according to the manufacturer’s protocol. Briefly, cells were seeded into six-well tissue culture plates in phenol red-free DMEM with 10% charcoal-stripped FBS at 37°C and 5% CO_2_ and treated with 0.1% DMSO or 25nM AF for 4, 24, 48, 72, or 120 hours. Cal51 cells were seeded into six-well tissue culture plates and treated with 0.1% DMSO or 250nM AF for 24, 48, 72, 120, or 168 hours. Triplicate samples were collected for all controls, and duplicate samples were collected for all treatment groups. Cells were collected, fixed, and stained for internal antigens according to manufacturer protocol. Samples were then analyzed on a BD LSRII. Data was analyzed using FlowJo version 9.6.4. Apoptosis and DNA damage in MDA-MB-468shAhR and Cal51shAhR was analyzed using immunofluorescence staining and western blot analysis of whole cell lysates.

### Senescence-associated β-galactosidase staining

Cal51shAhR cells were maintained in the presence of 0.1% DMSO or 250nM AF for nine days, or in the presence of 500nM of a known inducer of senescence, Doxorubicin (Doxo) for five days, in DMEM + 10% FBS at 37°C and 5% CO_2_. At the designated time points, triplicate samples were fixed in a 2% formaldehyde/0.2% glutaraldehyde solution for five minutes, and then stained overnight at 37°C with an X-Gal-containing staining buffer. After two PBS washes, samples were imaged at 10× on a Leica DM IL inverted microscope using the Leica Applications Suite software.

### Statistical analysis

DRE Luc data are expressed as mean ± S.E.M. Two-tailed, unpaired Student’s T Tests were performed for statistical analysis of DRE Luciferase data using Microsoft Excel, where * p ≤ 0.05 compared to DMSO control. qPCR data are expressed as mean expression ± corrected S.D. Three-parameter log versus inhibition nonlinear regression was performed for cell counting assays using GraphPad Prism Software (Version 5.04; Graph-Pad Software Inc., San Diego, CA). Cell cycle data is presented as mean percentage of cells ± S.D. Two-tailed, unpaired Student’s T Tests were performed for analysis of control versus treated samples to measure cell cycle alterations.

## Results

### ERα negative MDA-MB-468 and Cal51 human breast cancer cells exhibit sensitivity to aminoflavone

**Figure 1:**
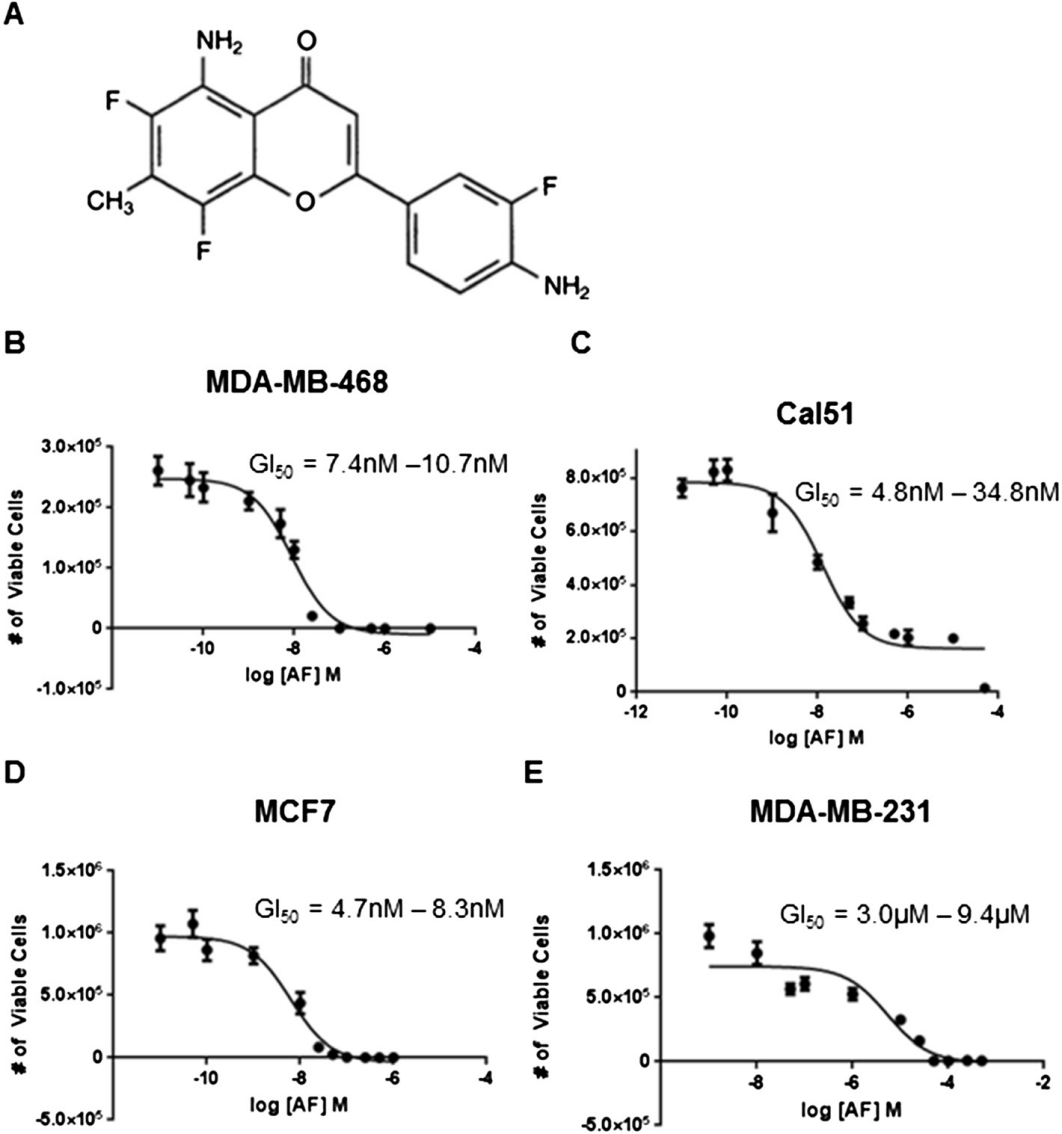
**ERα-negative MDA-MB-468 and Cal51 human breast cancer cell lines exhibit sensitivity to AF*. *(A)** Structure of Aminoflavone (5-amino-2-(4-amino-3-fluorophenyl)-6,8-difluoro-7-methylchromen-4-one; AF; NSC 686288). **(B)** GI_50_ (growth inhibition) mediated by AF plotted as concentration of AF in log [M] versus number of viable MDA-MB-468 cells. Cells were treated with AF for seven days. Data is presented as a 95% confidence interval of the GI_50_ value for AF. **(C)** GI_50_ (growth inhibition) mediated by AF plotted as concentration of AF in log [M] versus number of viable Cal51 cells. Cells were treated with AF for seven days. Data is presented as a 95% confidence interval of the GI_50_ value for AF. **(D)** MCF-7 human breast cancer cells and **(E)** MDA-MB-231 human breast cancer cells, which are reported to be sensitive and resistant respectively, were examined to validate the cell counting assay. Both cell lines were treated with AF for seven days.

We examined the expression of ERα and AhR in four human breast cancer cell lines (Additional file 1: Supplemental Methods; Additional file 2: Figure S1A, B). AhR was the lowest in MCF7 cells at both the protein (Additional file 2: Figure S1A) and mRNA level (Additional file 2: Figure S1B). In order to assess whether ERα expression is necessary for sensitivity to AF, we exposed MDA-MB-468 and Cal51, both ERα negative human breast cancer cell lines, to a range of AF concentrations (Figure 1A). MDA-MB-468 exhibited a 95% confidence interval of GI_50_ values between 7.4nM and 10.7nM (Figure 1B), and Cal51 exhibited a 95% confidence interval of GI_50_ values between 4.8nM and 34.8nM (Figure 1C). We confirm that MDA-MB-468 is sensitive to AF [23], while the finding that Cal51 is also exquisitely sensitive is novel. To validate this assay, MCF7, which has been reported to be sensitive to AF, and MDA-MB-231, which has been reported to be resistant, were assessed [8,17,19,20]. We confirmed AF sensitivity in MCF7 (Figure 1D), and insensitivity in MDA-MB-231 (Figure 1E). These results suggested that ERα expression may not be a determinant of AF sensitivity in all *in vitro* models, and may not be useful as a biomarker for responsiveness to this compound.

### Aminoflavone induces AhR-mediated expression of CYP1A1, CYP1B1, and luciferase downstream of dioxin responsive elements

**Figure 2:**
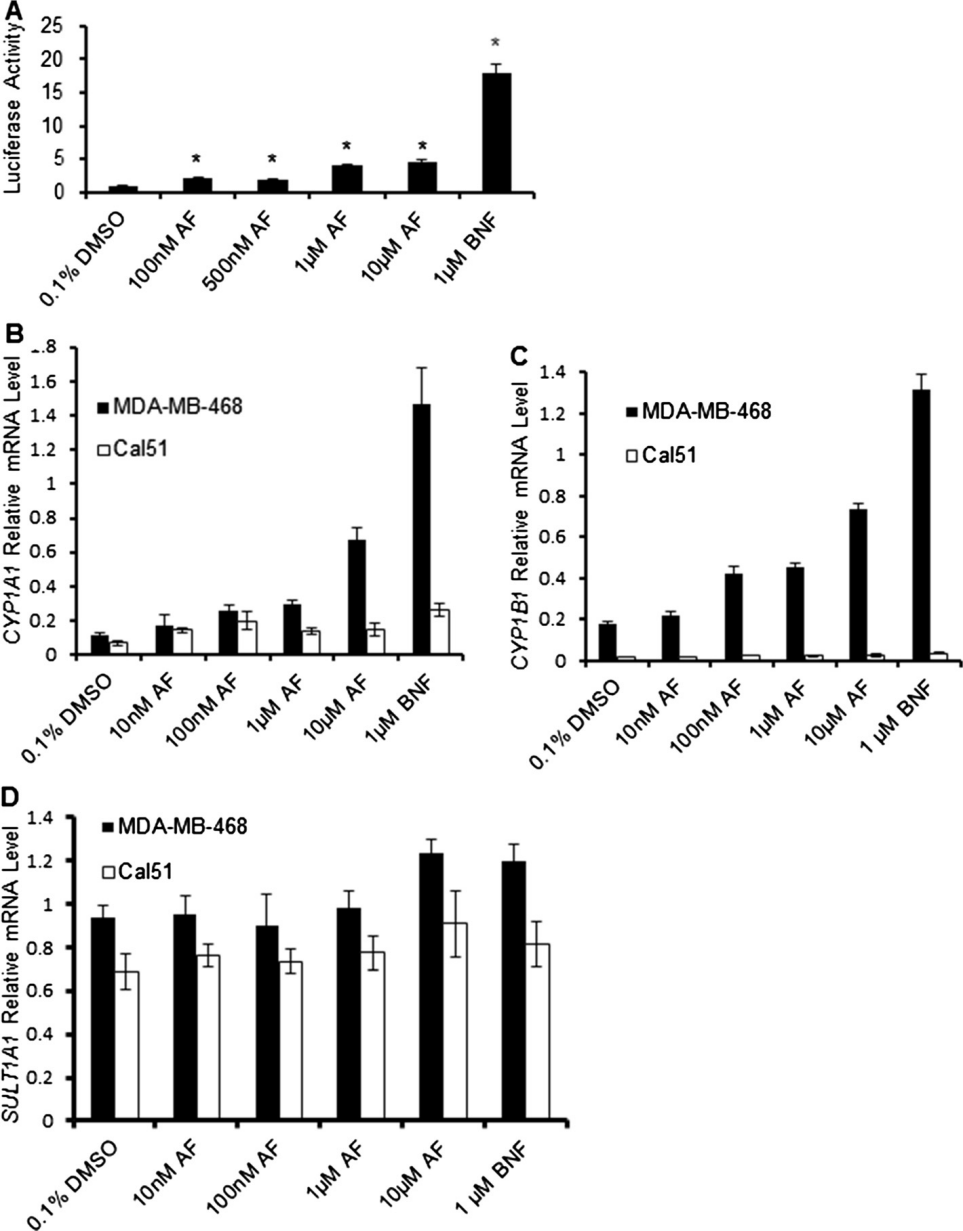
**AF increases expression of a DRE-luciferase reporter, CYP1A1, and CYP1B1. (A)** Quantitative representation of AF’s ability to induce luciferase expression downstream of DRE sites in the 101 L hepatoma model. Raw luciferase data was normalized to the DMSO control and to total protein in each sample as determined by the Bradford method. Data is presented as mean normalized luciferase activity ± S.E.M. of triplicate samples. * p ≤ 0.05 compared to DMSO control. **(B)** Quantitative representation of *RPL13A*-normalized levels of *CYP1A1* gene expression in MDA-MB-468 and Cal51 human breast cancer cell lines exposed to a range of AF concentrations and an AhR agonist as a positive control, using SYBR-based quantitative PCR. Data is presented as mean relative mRNA level ± S.D. of triplicate samples. **(C)** Quantitative representation of *RPL13A*-normalized levels of *CYP1B1* gene expression in MDA-MB-468 and Cal51 human breast cancer cell lines exposed to a range of AF concentrations, using SYBR-based quantitative PCR. Data is presented as mean relative mRNA level ± S.D. of triplicate samples. **(D)** Quantitative representation of *RPL13A*-normalized levels of *SULT1A1* gene expression in MDA-MB-468 and Cal51 human breast cancer cell lines exposed to a range of AF concentrations, using SYBR-based quantitative PCR. BNF serves as a positive control. Data is presented as mean relative mRNA level ± S.D.

To confirm the finding that AF is capable of activating AhR signaling, 101 L hepatoma cells stably harboring three dioxin responsive elements upstream of a luciferase reporter were incubated with 0.1% dimethyl sulfoxide (DMSO) or AF (100nM - 10 μM) for 18 hours. After normalizing raw luciferase units to the background levels seen in the DMSO control, we show that AF significantly increases luciferase expression in this system (Figure 2A). However, compared with the positive control, β-Naphthoflavone (BNF), it is evident that AF is a weak AhR agonist [39]. This result is consistent with the previous finding that AF has agonistic effects on AhR. Further, it was previously shown that AhR target genes *CYP1A1*, and to a lesser extent *CYP1A2* and *CYP1B1* are upregulated in response to AF treatment, and may play role in the metabolism of AF itself [7,8,17,19-21,25]. We went on to examine whether AF could induce AhR target genes in MDA-MB-468 and Cal51. Cells were treated with a range of AF concentrations from 10nM to 10 μM, along with 1 μM of BNF as a positive control for AhR activation [39]. While MDA-MB-468 and Cal51 exhibit similar sensitivities to AF based on their GI_50_ values, we found that their ability to upregulate *CYP1A1* and *CYP1B1* expression after AF treatment was drastically different. AF strongly induced *CYP1A1* (Figure 2B) and *CYP1B1* (Figure 2C) expression in MDA-MB-468, but to a much lesser extent in Cal51. Compared to MCF7, which has been shown to be responsive to AhR ligands, MDA-MB-468 exhibits greater induction of *CYP1A1* upon AhR activation (Additional file 2: Figure S1C). Cal51 exhibits greater induction of *CYP1A1* upon treatment with AhR activators as compared to MDA-MB-231, which is AF-resistant, but the induction is less than both MCF7 and MDA-MB-468 (Additional file 2: Figure S1C) [7,8,17,19,20,25]. *SULT1A1* expression has also been linked to AF sensitivity [20,21]. MDA-MB-468 and Cal51 cells express *SULT1A1* basally, but its expression is not induced by treatment with AF or BNF (Figure 2D). Further, we have shown that knocking down AhR does not decrease basal *SULT1A1* expression in MDA-MB-468, and only minimally alters *SULT1A1* expression in Cal51 (Additional file 3: Figure S2A, B). Interestingly, direct knockdown of SULT1A1 in these cell lines results in significantly increased resistance to AF’s cytotoxic effects (Additional file 1: Supplemental Methods; Additional file 3: Figure S2C-E). Overall, these results suggest that cell populations with varying ability to induce AhR signaling may exhibit AF sensitivity. Thus, active downstream AhR signaling may not be required to confer AF sensitivity.

### Endogenous levels of AhR are not required for sensitivity to aminoflavone in MDA-MB-468 and Cal51 human breast cancer cells

**Figure 3:**
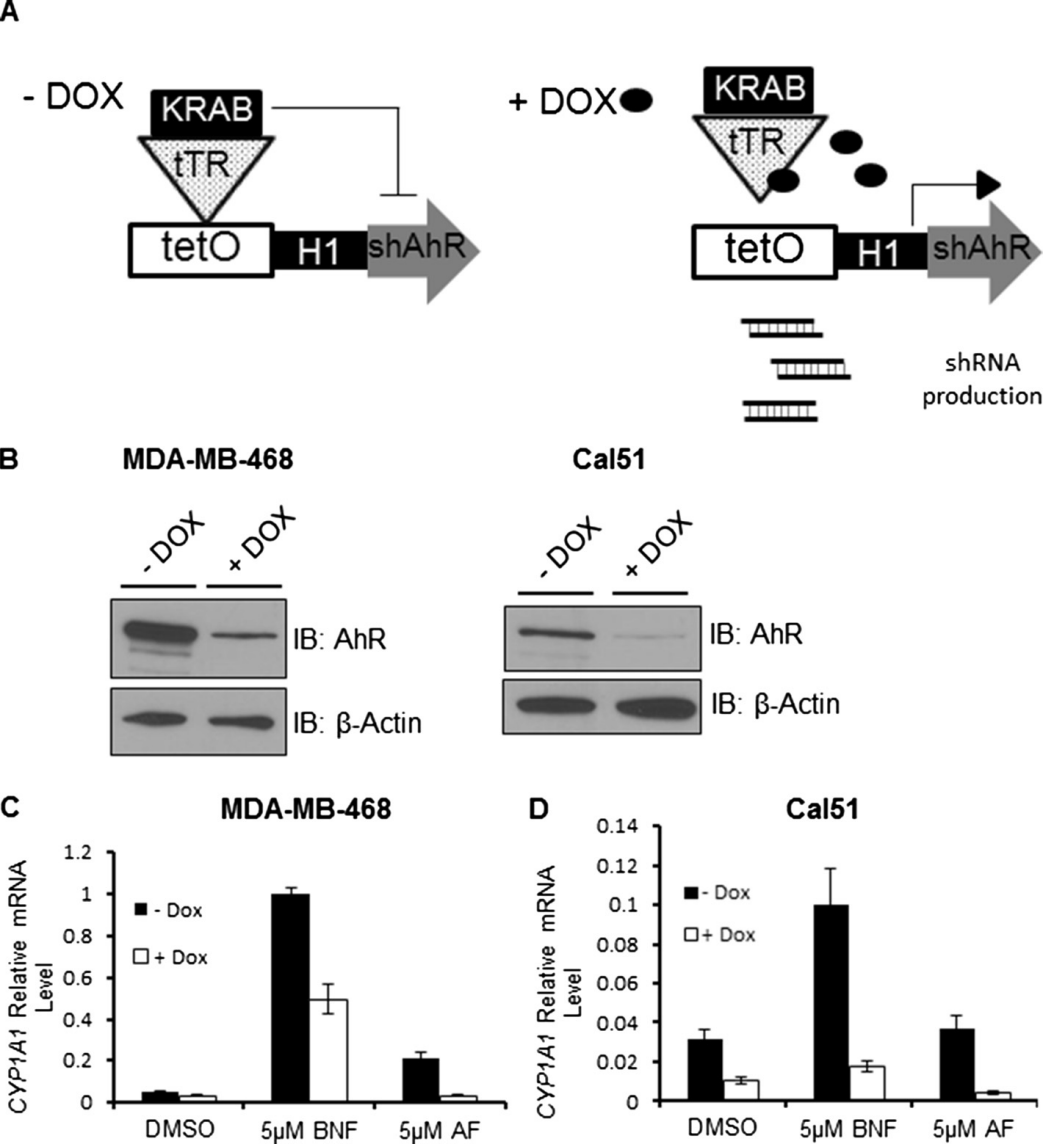
**AhR knockdown in MDA-MB-468 and Cal51 decreases AhR protein and expression of downstream targets*. *(A)** Model of Tet-On doxycycline (Dox)-inducible AhR knockdown system engineered in MDA-MB-468 and Cal51 human breast cancer cell lines (MDA-MB-468shAhR and Cal51shAhR). **(B)** Western blot of whole cell lysate from MDA-MB-468shAhR and Cal51shAhR treated with vehicle control or 750 ng/mL Dox in the tissue culture medium for 7 days. **(C)** Quantitative representation of *RPL13A*-normalized levels of *CYP1A1* gene expression in MDA-MB-468shAhR treated with vehicle control or 750 ng/mL Dox for seven days, then treated with compound for six hours. Data is presented as mean relative mRNA level ± S.D. of triplicate samples. **(D)** Quantitative representation of *RPL13A*-normalized levels of *CYP1A1* gene expression in Cal51shAhR treated with vehicle control or 750 ng/mL Dox for seven days, then treated with compound for six hours. Data is presented as mean relative mRNA level ± S.D. of triplicate samples.

It has been previously reported that AF-sensitive MCF7 cells become resistant to AF upon attenuation of AhR signaling. In addition, localization of AhR in the cellular cytoplasm has been shown to correlate with AF sensitivity [8,17,19,20]. As AhR may serve as a potential biomarker for sensitivity to AF, we examined the cellular localization as well as the requirement of endogenous levels of AhR for AF sensitivity in MDA-MB-468 and Cal51 cells. We showed using immunofluorescence that MDA-MB-468 and Cal51 cells express AhR in the cytoplasm, as well as strongly in the nucleus (Additional file 1: Supplemental Methods; Additional file 4: Figure S3). Using MDA-MB-468 and Cal51 harboring Dox-inducible AhR knockdown systems (Figure 3A), we repeated cell counting assays to determine the GI_50_ value of AF with and without knock down of endogenous AhR protein. To validate the ablation of the AhR pathway, we examined AhR protein level by western blot and *CYP1A1* induction after shRNA-mediated knockdown. Western blotting using whole cell lysate confirmed successful AhR knockdown after treating the cells with 750 ng/mL of Dox for seven days (Figure 3B). Correspondingly, *CYP1A1* induction by AF and BNF was attenuated in MDA-MB-468 (Figure 3C) and Cal51 (Figure 3D) after AhR knockdown by Dox treatment.

**Figure 4:**
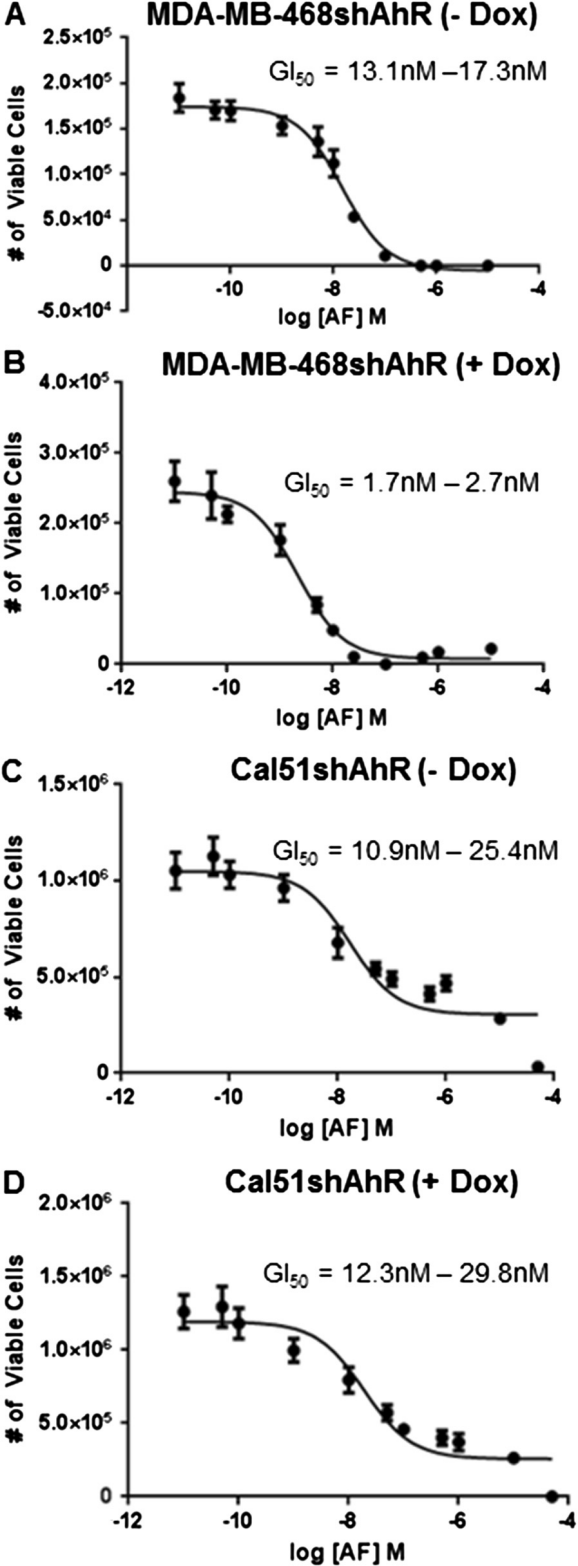
**AhR knockdown does not alter AF sensitivity in MDA-MB-468 and Cal51*. *(A)** GI_50_ (growth inhibition) mediated by AF plotted as concentration of AF in log [M] versus number of viable MDA-MB-468shAhR cells treated with vehicle control. All GI_50_ data is presented as a 95% confidence interval of the GI50 value for AF. **(B)** GI_50_ (growth inhibition) mediated by AF plotted as concentration of AF in log [M] versus number of viable MDA-MB-468shAhR cells treated with 750 ng/mL Dox in the tissue culture media for seven days prior to experiment plating (and maintained throughout the experiment). **(C)** GI_50_ (growth inhibition) mediated by AF plotted as concentration of AF in log [M] versus number of viable Cal51shAhR cells treated with vehicle control. **(D)** GI_50_ (growth inhibition) mediated by AF plotted as concentration of AF in log [M] versus number of viable Cal51shAhR cells treated with 750 ng/mL Dox in the tissue culture media for seven days prior to experiment plating (and maintained throughout the experiment). All cells were treated with AF for seven days.

As expected, MDA-MB-468shAhR (Figure 4A) and Cal51shAhR (Figure 4C) were sensitive to AF when endogenous levels of AhR protein are present, with GI_50_ ranges for AF of 13.1nM–17.3nM and 10.9nM–25.4nM, respectively. Similarly, when endogenous levels of AhR protein were decreased and AhR signaling was attenuated upon treatment with Dox, MDA-MB-468shAhR (Figure 4B) and Cal51shAhR (Figure 4D) exhibited GI_50_ values for AF ranging from 1.7nM–2.7nM and 12.3nM–29.8nM, respectively. We observed that the GI_50_ value for AF in MDA-MB-468shAhR decreases upon AhR knockdown. This may be attributed to variability in residual AhR levels post-knockdown. Further, because the concentrations of AF tested in this model reach as low as 0.01nM, variability in actual concentration may contribute to the apparent decrease. If AhR confers high sensitivity of cells to AF, knockdown of AhR is expected to increase GI_50_ value. However, AhR knockdown did not greatly affect AF sensitivity in either MDA-MB-468 or Cal51. These results suggest that an endogenous level of AhR protein is not responsible for high AF sensitivity in MDA-MB-468 and Cal51 human breast cancer cells. In addition, it supports our observation that a high level of AhR target gene induction does not necessarily predict sensitivity to AF. Given the incomplete knockdown of AhR by shRNA, we cannot exclude the possibility that residual AhR and AhR signaling post-knockdown is sufficient to sustain bioactivation of AF and confer AF sensitivity. In addition, AhR has been suggested to have extranuclear effects [40]. We have demonstrated that treatment with AF does not greatly modulate the phosphorylation of c-Jun in MDA-MB-468shAhR and Cal51shAhR cells, in the presence and absence of AhR knockdown (Additional file 1: Supplemental Methods; Additional file 5: Figure S4). These results suggest that AF sensitivity is not directly proportional to the endogenous level of AhR and the downstream activation of AhR in canonical and non-canonical ways.

### Low dose aminoflavone treatment results in differential mechanistic profiles in MDA-MB-468 and Cal51 human breast cancer cells

**Figure 5:**
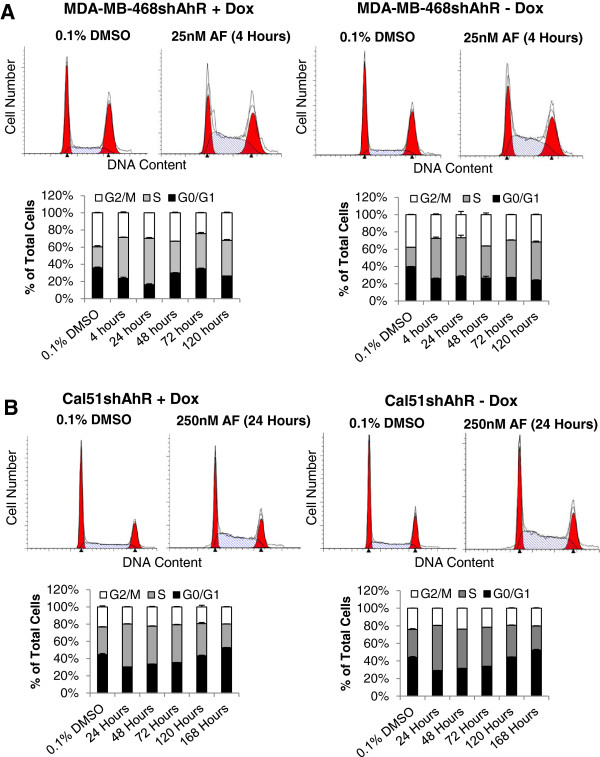
**AF induces cell cycle alterations in MDA-MB-468shAhR and Cal51shAhR. (A)** MDA-MB-468shAhR cells were pretreated with 750 ng/mL Dox or an equivalent amount of vehicle for seven days to induce AhR knockdown. Cells were then treated with 0.1% DMSO or 25nM AF, with or without co-treatment with 750 ng/mL of Dox, for the corresponding length of time. DNA content was evaluated using propidium iodide staining. A representative graph of DNA content versus cell number is shown for DMSO control (top left panel) and for the accumulation of cells in S phase (top right panel). All data in the top panels is presented as percentage of total cells in G0/G1, S, and G2/M phase for each treatment, shown with standard deviation. Statistical analysis in the form of Student’s *T*-test was used to compare the percentage of S phase cells between 0.1% DMSO-treated and AF-treated cells was performed, but not labeled due to the stacked nature of the graph **(B)** Cal51shAhR cells were pretreated with 750 ng/mL Dox or an equivalent amount of vehicle for seven days to induce AhR knockdown. Cells were then treated with 0.1% DMSO or 250nM AF, with or without co-treatment with 750 ng/mL of Dox, for the corresponding length of time. DNA content was evaluated using propidium iodide staining. A representative graph of DNA content versus cell number is shown for DMSO control (top left panel) and for the accumulation of cells in S phase (top right panel). All data in the right panel is presented as percentage of total cells in G0/G1, S, and G2/M phase for each treatment, shown with standard deviation. Statistical analysis in the form of Student’s *T*-test was used to compare the percentage of S phase cells between 0.1% DMSO-treated and AF-treated cells was performed, but not labeled due to the stacked nature of the graph.

**Figure 6:**
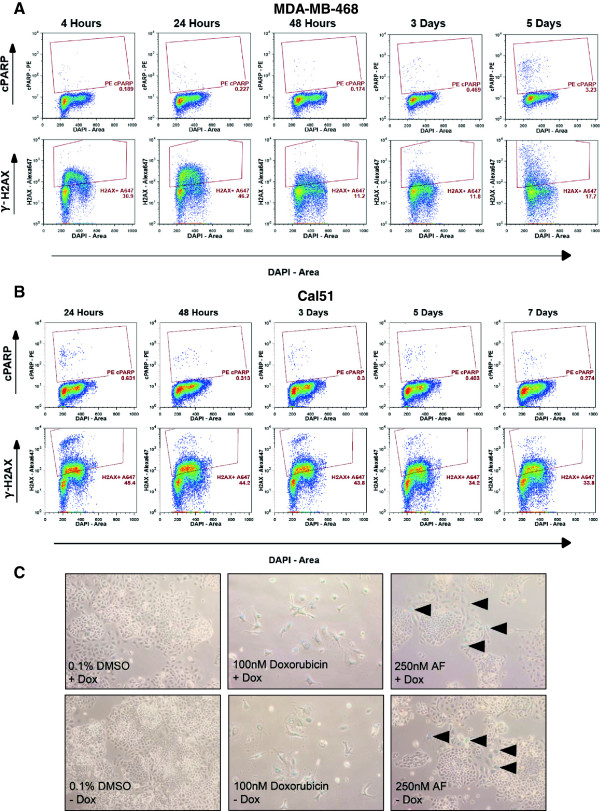
**AF induces DNA damage in MDA-MB-468 and Cal51, apoptosis in MDA-MB-468, and cellular senescence in Cal51shAhR.** The presence of γ-H2AX and cleaved PARP (cPARP) was evaluated using fluorescent antibody based flow cytometry in MDA-MB-468 **(A)** and Cal51 **(B)** cells. Cells were treated with 25nM AF (MDA-MB-468) or 250nM AF (Cal51) for the indicated periods of time and stained with the appropriate fluorescent antibody, per protocol requirements. Raw flow cytometry data is shown. Samples were run on a BD LSR II flow cytometer. Appropriate fluorescent minus one samples were used to gate and analyze sample data. **(C)** The presence of AF-induced cellular senescence in Cal51shAhR was examined by staining for senescence-associated β-Galactosidase. Cal51shAhR cells were pretreated with 750 ng/mL Dox or an equivalent amount of vehicle to induce AhR knockdown. Cells were then treated with 0.1% DMSO or 250nM AF (nine days) or 100nM of a known inducer of senescence, doxorubicin (five days), with or without co-treatment with 750 ng/mL Dox. Cells were then fixed and stained with an X-Gal-containing staining buffer. Images taken at 10x are shown.

A variety of mechanisms have been shown to underlie AF sensitivity in various cell types, including DNA-protein crosslinks, cytokeratin-RNA crosslinks, phosphorylation of p53, increased expression of p21, DNA damage, reactive oxygen species-mediated apoptosis, and S-phase arrest [7,8,17,19,20,22-25]. However, a majority of this work focused on ERα-positive, AF-sensitive cell populations, with the exception of one publication examining MDA-MB-468 [23]. After observing GI_50_ values for AF in the low nanomolar range for MDA-MB-468shAhR and Cal51shAhR, we chose to study the mechanism underlying AF sensitivity at relatively low concentrations. These concentrations (25nM AF for MDA-MB-468shAhR and 250nM for Cal51shAhR) were chosen based on the behavior of the cell lines in cell counting assays. Cal51shAhR exhibited static growth inhibition when treated with concentrations of AF greater than 100nM. For this reason, we chose to treat Cal51shAhR with 250nM AF. Using these concentrations, we examined cell cycle changes, senescence, DNA damage, and apoptosis. Upon treatment with 25nM AF, we observed an accumulation of MDA-MB-468shAhR cells in S phase beginning at 4 hours and lasting until 120 hours treatment, both in the presence and absence of AhR knockdown resulting from Dox treatement (Figure 5A). This increase in the percentage of cells in S phase was statistically significant compared to the control in all treated groups (p < 0.01). Cal51shAhR cells also exhibited an accumulation in S-phase upon treatment with 250nM AF, both in the presence and absence of AhR knockdown, but this arrest appeared to be reversed over the course of 168 hour (seven days) of treatment (Figure 5B). However, the increase in the percentage of cells in S phase was statistically significant at the level of p < 0.01 for the 24 hour, 48 hour, and 72 hour time points, and at the level of p < 0.05 at the 120 hour time point. There was no statistically significant increase in S phase cells at the 168 hour time point. To correspond to the observed S phase arrest (throughout the timecourse in MDA-MB-468shAhR, and up until the 120 hour time point in Cal51shAhR), we demonstrated an accumulation of Cyclin A2, which is synthesized at the onset of DNA synthesis, in response to treatment with 25nM and 250nM for MDA-MB-468shAhR and Cal51shAhR respectively [41] (Additional file 1: Supplemental Methods; Additional file 6: Figure S5A, B). To examine the underlying mechanism of AF-mediated growth arrest, we used flow cytometry to analyze levels of the DNA damage marker, phosphorylated H2AX at serine 139 (γ-H2AX), as well as levels of cleaved poly-ADP ribose polymerase (PARP), which is a marker of apoptosis, in MDA-MB-468 and Cal51 parental cells. γ-H2AX was found to be elevated in MDA-MB-468 cells as early as four hours of treatment with 25nM AF (Figure 6A). Further, low dose AF treatment resulted in an increase in PARP cleavage after five days (Figure 6A, Additional file 1: Supplemental Methods; Additional file 6: Figure S5C). An approximate six to nine fold increase in γ-H2AX resulted from treatment with 250nM AF in Cal51 for the duration of the compound treatment, but presence of PARP cleavage was not evident (Figure 6B). To more thoroughly examine the kinetics of γ-H2AX in response to AF, we stained γ-H2AX foci in MDA-MB-468shAhR and Cal51shAhR by immunofluorescence, both in the presence and absence of AhR knockdown. In these models, we performed AF dose response, timecourses of 25nM AF and 250nM AF for MDA-MB-468shAhR and Cal51shAhR respectively, and recovery experiments subsequent to treatment with these concentrations of AF. γ-H2AX stained cells were qualitatively analyzed for light staining, discrete foci, or diffuse staining [22]. We did not observe a significant dependency of DNA damage on AF dose, as γ-H2AX staining was consistently high at low and high concentrations of AF in both cell models in the presence and absence of AhR knockdown (Additional file 1: Supplemental Methods; Additional file 7: Figure S6). We also did not observe a significant dependency of DNA damage on the length of AF treatment. γ-H2AX staining increased at the earliest time points in both cell models in the presence and absence of AhR knockdown, and they remained high throughout the timecourse (Additional file 1: Supplemental Methods; Additional file 8: Figure S7). Further, it did not appear that γ-H2AX in response to AF treatment was reversible in MDA-MB-468shAhR and Cal51shAhR at 25nM and 250nM respectively, both in the presence and absence of AhR knockdown (Additional file 1: Supplemental Methods; Additional file 9: Figure S8). Lastly, we found that treatment of Cal51shAhR with 250nM of AF for nine days induced the presence of senescence-associated β-galactosidase expression, both in the presence and absence of AhR knockdown (Figure 6C). These results showed that AF-mediated growth inhibition may occur through varying mechanisms. While DNA damage and S-phase cell cycle arrest occurred in both MDA-MB-468 and Cal51 cells, the apoptotic response appeared to occur in only MDA-MB-468, and a senescent-like phenotype was only observed in Cal51.

## Discussion

AF is a novel anticancer drug candidate that had been investigated in multiple clinical trials, although the biomarker (s) predictive of AF anticancer activity have not been defined.

Numerous studies have investigated the effects of AF treatment in human tumor cell lines, as well as the mechanisms underlying sensitivity and the effects in combination with other anticancer drugs [42]. However, the main body of work focuses on a few model cell lines, in particular, AF-sensitive ERα-positive MCF7. While there seems to be a correlation between ERα expression and AF sensitivity in the NCI 60 cell line screen and the literature, it is imperative to fully explore the properties of sensitive populations of cells to discover potential biomarker (s) for patient stratification in clinical trials. For example, one publication suggests that ERα, while an indicator of AF sensitivity, may not be a reliable predictor of AF effectiveness in all cases, as ERα-negative MDA-MB-468 human breast cancer cells also exhibited sensitivity [23]. MDA-MB-231 and MDA-MB-453 are commonly used to demonstrate insensitivity to AF in ERα negative human breast cancer cell lines. Given the poor clinical prognosis and lack of targeted therapies associated with triple negative breast cancers, examining a wider range of ERα-negative breast cancer cell lines to understand AF’s effects is important.

Recent studies draw attention to the relationship between AF sensitivity and AhR signaling. AF was shown to be an agonist of AhR signaling, and AF-induced growth inhibition in renal and breast cancer cells is mediated by AhR activation [7,8,17,19,20,22-25]. Our results are consistent with the finding that AF is able to induce AhR signaling. We showed that AF could activate a DRE-driven luciferase reporter and induce expression of *CYP1A1* and *CYP1B1* in AF-sensitive, ERα-negative MDA-MB-468 human breast cancer cells. Interestingly, we found that AF sensitivity could be uncoupled from AhR responsiveness as exemplified in a human breast cancer cell line, Cal51. We first showed that Cal51 cells, while expressing high levels of endogenous AhR protein, lack *CYP1A1* and *CYP1B1* induction upon treatment with AhR activators. Cal51 cells are sensitive to AF, exhibiting a GI_50_ value in the nanomolar range. When AhR is knocked down in Cal51shAhR, inducibility of *CYP1A1* was further attenuated, yet AF’s GI_50_ value was not greatly affected. MDA-MB-468 cells are relatively responsive to AhR activation, and like Cal51, they maintain sensitivity to AF after AhR knockdown. SULT1A1 has also been implied in the bioactivation of AF, and we showed that both MDA-MB-468 and Cal51 express a basal level of *SULT1A1* mRNA. However, AF and BNF were unable to increase levels of this gene. The AhR-independency of AF sensitivity in MDA-MB-468 and Cal51 is in discrepancy with the finding that MCF7 cells are sensitive to AF while Ah^R100^ MCF7 cells are AF resistant. Previous studies had shown that Ah^R100^ cells exhibited diminished AhR protein levels, mRNA levels, and ability to induce AhR target genes, rendering the cell line resistant to AF [8,18,19]. The difference in AF sensitivity with regards to AhR levels and activity could be cell type specific. Nonetheless, our data from parental Cal51 as well as from Cal51shAhR and MDA-MB-468shAhR provide strong evidence that AhR protein level, as well as downstream AhR signaling may not be directly predictive of AF sensitivity in all cell types. While our data suggests that the growth inhibitory effects of AF still occur in cells where the levels of AhR and AhR signaling are significantly decreased, we have only examined the genomic activity of AhR in the context of AF signaling. It is important to note that AhR has been shown to have non-genomic kinase activity, including interactions with Src and effects on Ca^2+^ as a second messenger in inflammatory pathways [40], [43]. The potential role of AhR’s extranuclear effects in the context of AF sensitivity has yet to be uncovered.

While MDA-MB-468 and Cal51 human breast cancer cell lines exhibit similar GI_50_ values for AF, graphing the log of AF concentration versus viable cell number shows that the two curves differ in shape. In the graphical representation of growth inhibition by AF in MDA-MB-468, we see that higher concentrations of AF can eliminate viable cells completely. In contrast, some Cal51 cells can still survive even at the highest AF concentrations. Due to the different profiles of their GI_50_ graphs, we predicted that the mechanisms underlying AF-mediated growth inhibition would vary.

Because the growth inhibition mediated by AF in Cal51 plateaued at a positive number of viable cells as concentration increased (with the exception of the highest concentration of 50 μM), we predicted that AF had a cytostatic effect on the cells without immediately inducing cell death. Indeed, we have reported the first instance of low dose (250nM) AF treatment resulting in cellular senescence, as shown using senescence-associated β-galactosidase staining, in Cal51shAhR human breast cancer cells, both in the presence and absence of AhR knockdown. However, it has been proposed that the permanent and irreversible arrest characteristic of senescence is a tumor suppressing mechanism, and must be overcome for tumorigenesis and immortalization of tumor cell lines [44]. The Cal51 human breast cancer cell line is extremely interesting in that it is tumorigenic, yet it consists of a population of cells that are identified by a stable and normal karyotype [31]. This cell line is to our knowledge, the only human breast cancer cell line carrying a normal karyotype. It remains to be determined whether induction of cellular senescence by AF is linked to normal karyotype. It has been shown that different genetic abnormalities are present in three TNBC cell lines with differing AF sensitivities [45]. MDA-MB-231 (PTEN wild type, p53 mutant, BRCA1 wild type) is resistant to AF. MDA-MB-468 (PTEN null, p53 mutant, BRCA1 wild type) and Cal51 (PTEN null, p53 wild type, BRCA1 wild type) are sensitive to AF. A previous study had shown that MDA-MB-468 and Cal51 cells are more susceptible to the cytotoxic effects of several PARP inhibitors than MDA-MB-231 cells [46]. Whether the common cytotoxicity of PARP inhibitors and AF in MDA-MB-468 and Cal51 are linked to their shared PTEN and BRCA1 status warrants further investigation.

AF induces DNA damage in both MDA-MB-468 and Cal51 cell lines, both parental and AhR knockdown cell lines, which is consistent with previous findings [23]. We observed an increase in γ-H2AX in MDA-MB-468 treated with 25nM AF as early as 4 hours using flow cytometry (Figure 6A). We also observed DNA damage in MDA-MB-468shAhR using immunofluorescence staining for γ-H2AX in the presence and absence of AhR knockdown (Additional file 7: Figure S6A, Additional file 8: Figure S7A, Additional file 9: Figure S8A). Extensive DNA damage was observed in Cal51 treated with 250nM AF as shown by flow cytometry, and the same was shown for Cal51shAhR using immunofluorescence staining for γ-H2AX (Figure 6B, Additional file 7: Figure S6B, Additional file 8: Figure S7B, Additional file 9: Figure S8B). In MDA-MB-468shAhR and Cal51shAhR, both in the presence and absence of AhR knockdown, we observed that the DNA damage response (using γ-H2AX as a marker) occurred at low concentrations and early time points, and was irreversible at 8 hours post-removal of AF. Cal51 has been found to display microsatellite instability as well as mutation is mismatch repair genes, offering a potential explanation for this apparent lack of DNA repair [47]. While DNA damage occurs in both cell lines, an apoptotic response was only observed in AF-treated MDA-MB-468.

## Conclusions

In summary, we showed that MDA-MB-468 and Cal51, both ERα negative human breast cancer cell lines, are sensitive to growth inhibition mediated by AF. This growth inhibition occurs regardless of whether or not the cells are induced by doxycycline to decrease AhR protein levels significantly and attenuate genomic AhR signaling. While low-dose AF induced DNA damage and S-phase arrest in both MDA-MB-468shAhR and Cal51shAhR, AF caused apoptosis in MDA-MB-468shAhR, and a senescent-like phenotype in Cal51shAhR. Our results suggest that AF may be a viable therapeutic option for broader subtypes of breast cancers. While the underlying mechanism of AF-mediated growth inhibition may vary between cell lines, and likely between individual tumors, it is encouraging that AF, even at very low doses, is effective in more than one TNBC cell line. At present, chemotherapy is the only available treatment for TNBC. Given that systemic toxicity is a recurring problem in chemotherapies, and is also the cause of suspension for several Phase I and II clinical trials for AF, this work suggests that further studies are needed to identify potential biomarkers to stratify patient populations that might benefit from low dose AF treatment to circumvent systemic toxicity.

## Abbreviations

AF: Aminoflavone; AhR: Aryl hydrocarbon receptor; ARNT: Aryl hydrocarbon receptor nuclear translocator; BNF: β-naphthoflavone; CYP: Cytochrome P450; DMEM: Dulbecco’s modified eagle’s medium; DMSO: Dimethyl sulfoxide; DOXO: Doxorubicin; Dox: Doxycycline; DRE: Dioxin responsive element; ER: Estrogen receptor; FBS: Fetal bovine serum; GI50: Growth inhibition value; HER2: Human epidermal growth factor receptor 2; PARP: Poly-ADP ribose polymerase; γ-H2AX: Phosphorylated histone 2AX; PR: Progesterone receptor; PI: Propidium iodide; qPCR: Quantitative PCR; shRNA: Small hairpin RNA; SULT1A1: Sulfotransferase 1A1; TNBC: Triple negative breast cancer.

## Competing interests

The authors declare that they have no competing interests.

## Authors’ contributions

Experimental design (AMB, JW, KE, WX); Data collection (AMB, KE); Data analysis (AMB, KE); Figure preparation (AMB, KE, WX); Manuscript writing (AMB, WX). All authors read and approved the final manuscript.

## Pre-publication history

The pre-publication history for this paper can be accessed here:

http://www.biomedcentral.com/1471-2407/14/344/prepub

## Supplementary Material

Additional file 1 Supplemental Methods. Click here for file

Additional file 2: Figure S1 *Expression of AhR, ER*α*, and CYP1A1 in MDA-MB-468, MDA-MB-231, Cal51, and MCF7 human breast cancer cells.* (A). Whole cell lysates were collected from MDA-MB-468, MDA-MB-231, Cal51, and MCF7 human breast cancer cells. Western blotting indicates the relative protein expression levels of AhR and ERα in these cell lines. (B). Total RNA was collected from MDA-MB-468, MDA-MB-231, Cal51, and MCF7 human breast cancer cells and reverse transcribed. qPCR was performed for *AHR* and *ESR1* (ERα) transcript, and the data is shown as mean relative mRNA level normalized to *RPL13A*, ± S.D of triplicate values. (C). Total mRNA was collected from MDA-MB-468, MDA-MB-231, Cal51, and MCF7 human breast cancer cells treated with 0.1% DMSO, 1 μM AF, or 1 μM BNF for 6 hours, and reverse transcribed. qPCR was used to determine the induction of the *CYP1A1* gene (normalized to *RPL13A)*, shown as ± S.D of triplicate values. Click here for file

Additional file 3: Figure S2 *AhR knockdown results in minimal alteration of SULT1A1 expression in MDA-MB-468shAhR and Cal51shAhR cells, while an efficient knockdown of SULT1A1 results in an increase in resistance to cytotoxicity mediated by AF.* Total RNA was collected from MDA-MB-468shAhR (A) and Cal51shAhR (B) cells pretreated with 750 ng/mL Dox or vehicle for seven days to induce AhR knockdown, and subsequently treated with 0.1% DMSO, 5 μM BNF, or 5 μM AF for six hours. qPCR was performed for *SULT1A1*, and the data is shown as mean relative mRNA level normalized to *RPL13A* ± S.D. of triplicate values. *SULT1A1* expression is minimally effected by AhR knockdown. (C). Total RNA was collected from parental MDA-MB-468 and Cal51 cells infected with lentivirus containing a scrambled shRNA or shRNA directed toward SULT1A1. qPCR was performed for *SULT1A,* and the data is shown as mean relataive mRNA level normalized to *RPL13A* ± S.D. of triplicate values. *SULT1A1* knockdown appears to be efficient at the transcript level. 3-[4,5-dimethylthiazol-2-yl]-2,5 diphenyl tetrazolium bromide (MTT) assays were performed in (D) MDA-MB-468 cells harboring SULT1A1 shRNA and (E) Cal51 cells harboring SULT1A1 shRNA. Cells were plated in a 96-well format and treated with 0.1% DMSO or varying concentrations of AF for 48 hours prior to incubation with MTT. Knockdown of SULT1A1 results in enhanced resistance to AF-mediated cytotoxicity. **p < 0.01, *p < 0.05. Click here for file

Additional file 4: Figure S3 *AhR is localized to both the cytoplasm and nucleus in Cal51 and MDA-MB-468 human breast cancer cells.* Immunofluorescence for AhR was performed in Cal51 and MDA-MB-468, showing that AhR localizes to the cytoplasm, but also strongly in the nuclei of these cells. Images were acquired at 40×. Click here for file

Additional file 5: Figure S4 *AF does not have significant effects on JNK activity in MDA-MB-468shAhR and Cal51shAhR cells.* (A)**.** Whole cell lysates were collected from MDA-MB-468shAhR pretreated with 750 ng/mL Dox and subsequently treated with 25nM AF, in the presence and absence of AhR knockdown by maintaining 750 ng/mL Dox or vehicle in the media. Western blotting shows that compared total c-Jun levels, phosphorylated c-Jun (p-c-Jun) does not appear to be affected by AF treatment. (B)**.** Whole cell lysates were collected from Cal51shAhR pretreated with 750 ng/mL Dox and subsequently treated with 250 nM AF, in the presence and absence of AhR knockdown by maintaining 750 ng/mL Dox or vehicle in the media. Western blotting shows that compared total c-Jun levels, phosphorylated c-Jun (p-c-Jun) does not appear to be affected by AF treatment. We observe a decrease of total c-Jun protein at the 7 day time point. HSP90 was used as a loading control. Click here for file

Additional file 6: Figure S5 *Cyclin A2 increases in response to AF treatment in MDA-MB-468shAhR and Cal51shAhR cells.* Whole cell lysates were collected from MDA-MB-468shAhR (A) and Cal51shAhR (B) pretreated with 750 ng/mL Dox and subsequently treated with 25nM AF or 250nM AF respectively, in the presence and absence of AhR knockdown by maintaining 750 ng/mL Dox or vehicle in the media. Western blotting shows that compared to control, AF causes an increase in Cyclin A2 protein in MDA-MB-468shAhR during the timecourse, both in the presence and absence of AhR knockdown, consistent with the observed S-phase cell cycle arrest. Cyclin A2 protein levels initially increase in Cal51shAhR, then decrease at the end of the timecourse, both in the presence and absence of AhR knockdown. This is consistent with the S-phase arrest observed in cell cycle analysis, with the 7 day (168 hour) time point having no statistically significant increase in percentage of S-phase cells. (C). Whole cell lysates were collected from MDA-MB-468shAhR pretreated with 750 ng/mL Dox and subsequently treated with 25nM AF, in the presence and absence of AhR knockdown by maintaining 750 ng/mL Dox or vehicle in the media. Western blotting shows that after 48 hours, 25nM AF causes PARP cleavage. Click here for file

Additional file 7: Figure S6 *The intensity of* γ*-H2AX staining is not proportional to AF dose in MDA-MB-468shAhR and Cal51shAhR cells.* MDA-MB-468shAhR (A) and Cal51shAhR (B) were treated with a range of AF concentrations and then subjected to immunofluorescence staining for γ-H2AX. FITC (γ-H2AX) images were overlaid upon DAPI (nuclear), and at least thirty individual cells were assessed for intensity of γ-H2AX staining. We observed that γ-H2AX staining that remained constant regardless of AF dose. Click here for file

Additional file 8: Figure S7 γ*-H2AX staining intensity is not time dependent in MDA-MB-468shAhR and Cal51shAhR cells.* MDA-MB-468shAhR (A) and Cal51shAhR (B) were treated with 25nM or 250nM AF respectively for six hours, then subjected to immunofluorescence staining for γ-H2AX. FITC (γ-H2AX) images were overlaid upon DAPI (nuclear), and at least thirty individual cells were assessed for intensity of γ-H2AX staining. We observed that γ-H2AX staining remained relatively constant over the timecourse. Click here for file

Additional file 9: Figure S8 γ*-H2AX staining intensity is not reversed by the removal of AF for eight hours in MDA-MB-468shAhR and Cal51shAhR cells.* MDA-MB-468shAhR (A) and Cal51shAhR (B) were treated with 25nM and 250nM AF respectively for six hours, then was replaced with untreated media for various lengths of time. Samples were subjected to immunofluorescence staining for γ-H2AX. FITC (γ-H2AX) images were overlaid upon DAPI (nuclear), and at least thirty individual cells were assessed for intensity of γ-H2AX staining. We observed that even after eight hours after AF removal, γ-H2AX staining persists, indicating that DNA damage mediated by AF may be irreversible in these cell lines. Click here for file
