# Supplementary material for: Estrogen receptor α and aryl hydrocarbon receptor independent growth inhibitory effects of aminoflavone in breast cancer cells
Source: BMC Cancer. 2014 May 20;14:344. doi: 10.1186/1471-2407-14-344 (PMC4037283; doi:10.1186/1471-2407-14-344)
Supplement: Additional file 1 — Supplemental Methods. [file 1471-2407-14-344-S1.docx]

**Brinkman et al., Additional File 1 – Supplemental Methods**

**Supplemental Methods**

**Gene Expression Analysis for *AHR and ESR1***

MDA-MB-468 and Cal51, cells were cultured in phenol red-free DMEM + 10% charcoal stripped FBS at 37⁰C and 5% CO_2_ for three days prior to experiment to remove residual estrogens. Triplicate 80% confluent six cm tissue culture dishes of MDA-MB-468 and Cal51 were collected. Total RNA was extracted using HP Total RNA Kit (VWR Scientific, West Chester, PA) according to the manufacturer’s protocol. Two micrograms of RNA were reverse transcribed using Superscript II RT according to the manufacturer’s protocol (Invitrogen). Fast Start Universal SYBR Green Master Mix (Roche) was used to perform qPCR for *AHR* and *ESR1* (which encodes for ERα protein) on a BioRad CFX-96 instrument, using *RPL13A* as a housekeeping gene (BioRad). The primer sequences are as follows: *ESR1* For 5’ GCGCTCGAGGGGCCACCTTTAGCAGATC 3’, *ESR1* Rev 5’ GCGAAGCTTCAGGGTGCAGACCGTGTC 3’, *AHR* For 5’ TCCACCTCAGTTGGCTTTGTTTGC 3’, *AHR* Rev 5’ TCGTGCACAGCTCTGCTTCAGTAT 3’.

**Knockdown of SULT1A1 by Lentiviral Infection**

MDA-MB-468 and Cal51 cells cultured in DMEM + 10% FBS were seeded into six-well plates to reach a confluency of 50-60% the subsequent day and incubated overnight at 37⁰C and 5% CO_2_. Media was then supplemented with 5μg/mL polybrene and ~30uL of packaged lentiviral particles containing either control shRNA or SULT1A1 shRNA (Santa Cruz sc-108080, Santa Cruz sc-106578-V) from a viral stock of 1x10^6^ infection units (IFU) of virus. Viral particles were incubated on the cells at 37⁰C and 5% CO_2_ for 18 hours. Media containing virus was then removed and replaced with DMEM + 10% for 24 hours. Cells were passaged appropriately, and on the second day post-transduction, media was supplemented with 2 μg/mL puromycin to begin pooling shRNA-containing cells. Puromycin was applied for 4 days, and remaining cells were used for downstream analysis.

**3-[4,5-dimethylthiazol-2-yl]-2,5 diphenyl tetrazolium bromide (MTT) Assay**

MDA-MB-468 and Cal51 cells containing either control shRNA or SULT1A1 shRNA were seeded at a density of 4500 cells/well or 3000 cells/well, respectively, in DMEM + 10% FBS using duplicate 96-well plates. Cells were allowed to attach for six hours at 37⁰C and 5% CO_2_, and were then treated with 0.1% DMSO or a range of AF concentrations in DMEM + 10% FBS. Each treatment condition contained at least five replicate wells. Cells were incubated with treatment for 48 hours at 37⁰C and 5% CO_2_. After 48 hours, treatment was removed and cells were washed three times with warm 1X PBS. DMEM + 10% FBS supplemented with 500μg/mL MTT was added to the wells and incubated at 37⁰C and 5% CO_2_ for three hours. After incubation with MTT, media was removed from the wells and replaced with 200μL DMSO. Plates were placed on a rotating mixer for five minutes, and then absorbance at 595nm (peak absorbance) and 650nm (background cell absorbance) using a PerkinElmer Victor X5 multilabel plate reader and the corresponding software. Background absorbance was subtracted from peak absorbance, and data was normalized as a percentage of the 0.1% DMSO-treated samples.

**Immunofluorescence**

Cal51 and MDA-MB-468 cells were seeded at 100,000 cells/well in a six-well tissue culture plate containing one round glass coverslip per well using DMEM with 10% FBS. After attachment, coverslips and the attached cells were washed with PBS and fixed with 4% paraformaldehyde for ten minutes at room temperature. After fixation, coverslips were washed three times with PBS. Cells were permeabilized using 0.2% Triton X-100 in PBS for three minutes, followed by three PBS washes. Coverslips were blocked using a 10% solution of goat serum in PBS for one hour at room temperature. A 1:250 solution of anti-AhR antibody (Santa Cruz, sc-5579) was prepared in 2% BSA, and coverslips were incubated with antibody for one hour at room temperature, followed by three PBS washes. A 1:250 solution of FITC-conjugated secondary antibody (Jackson Immunoresearch, 111-095-144) was prepared in 2% BSA, and coverslips were incubated for one hour at room temperature, protected from light. Coverslips were washed three times with PBS, and mounted with Prolong Gold containing DAPI (Invitrogen, P36931). Images were acquired using the 40X objective on a Leica DM 5000 B upright fluorescent microscope using the Leica Applications Suite software.

**Immunoblotting for c-Jun/p-c-Jun, PARP, Cyclin A2, ERα, HSP90, or β-Actin**

MDA-MD-468shAhR and Cal51shAhR were pretreated for seven days with vehicle or 750ng/mL doxycycline (Dox) in DMEM with 10% Tet-Approved FBS. Cells were then seeded into 6 centimeter tissue culture dishes and treated with either 0.1% DMSO, 25nM AF (MDA-MB-468shAhR), or 250nM AF (Cal51shAhR) for the appropriate length of time. After treatment, cells were collected by trypsinization, washed with 1x PBS (Gibco, Invitrogen), and lysed using Triton X-100 lysis buffer (50 mM Tris pH 8.0, 400 mM NaCl, 10% glycerol, 0.5% triton X-100, protease inhibitors, and benzonase). Total protein concentration was measured using the Bradford method (BioRad), and 20μg of protein was resolved using SDS-PAGE on 8% polyacrylamide gels. Protein was transferred to a nitrocellulose membrane at 4⁰C for one hour at 0.35A. Membranes were blocked with 5% nonfat milk in PBS + 0.1% Tween for one hour at room temperature, then incubated with either 1:3000 anti-HSP90 antibody (Santa Cruz, sc-7947), 1:10,000 anti-β-Actin antibody (Sigma, A5316), 1:1000 anti-c-Jun antibody (Santa Cruz, sc-1694), 1:1000 anti-p-c-Jun (Ser 63/73) antibody (Santa Cruz, sc-16312-R), 1:500 anti-Cyclin A2 antibody (Cell Signaling 4656-P), 1:3000 anti-ERα antibody (Santa Cruz, sc-543), or 1:100 anti-PARP antibody (Cell Signaling, 9542S). Membranes were incubated with 1:10,000 goat anti-rabbit HRP or anti-mouse HRP secondary antibody for one hour at room temperature. Enhanced chemiluminescence reagents (Thermo Scientific) were applied to the membranes prior to exposure to x-ray film (Kodak).

**Analysis of γ-H2AX Kinetics**

MDA-MB-468shAhR and Cal51shAhR cells were seeded at 250,000 cells/well in a six-well tissue culture plate containing one round glass coverslip per well in DMEM with 10% Tet-Approved FBS and allowed to attach overnight at 37⁰C and 5% CO_2_. One group of cells was then treated with 0.1% DMSO, 25nM AF (MDA-MB-468shAhR only), 250nM AF (Cal51shAhR only), 1μM AF, or 5μM AF for six hours at 37⁰C and 5% CO_2_. A second group of cells was treated with 0.1% DMSO, 25nM AF(MDA-MB-468shAhR) or 250nM AF (Cal51shAhR) for either 0.5 hour, 1 hour, 3 hours, 6 hours, 12 hours, 18 hours, or 24 hours at 37⁰C and 5% CO_2_. A third group of cells was treated with 25nM AF (MDA-MB-468shAhR) or 250nM AF (Cal51shAhR) for 6 hours, then AF-containing media was removed and replaced with untreated media for 0 hour, 1 hour, 4 hours, or 8 hours. At the appropriate time points, the cells were washed with PBS and fixed with 4% paraformaldehyde for ten minutes at room temperature. After fixation, coverslips were washed three times with PBS. Cells were permeabilized using 0.2% Triton X-100 in PBS for three minutes, followed by three PBS washes. Coverslips were blocked using a 10% solution of normal donkey serum in PBS for one hour at room temperature. A 1:500 solution of anti-γ-H2AX antibody (Millipore, #05-636) was prepared in 2% BSA, and coverslips were incubated with antibody for one hour at room temperature, followed by three PBS washes. A 1:500 solution of FITC-conjugated secondary antibody (Jackson Immunoresearch, 795-095-151) was prepared in 2% BSA, and coverslips were incubated for one hour at room temperature, protected from light. Coverslips were washed three times with PBS, and mounted with Prolong Gold containing DAPI (Invitrogen, P36931). Images were acquired using the 100X objective on a Leica DM 5000 B upright fluorescent microscope using the Leica Applications Suite software. Three separate fields were imaged for each sample.γ-H2AX (FITC) staining was overlaid upon DAPI (nuclear) staining using Adobe Photoshop CS4, and individual cells were qualitatively assessed as having light γ-H2AX staining, discrete γ-H2AX foci, or diffuse γ-H2AX staining. At least thirty individual cells were analyzed per treatment group.
