# Supplementary material for: Estrogen receptor α and aryl hydrocarbon receptor independent growth inhibitory effects of aminoflavone in breast cancer cells
Source: BMC Cancer. 2014 May 20;14:344. doi: 10.1186/1471-2407-14-344 (PMC4037283; doi:10.1186/1471-2407-14-344)
Supplement: Additional file 2: Figure S1 — Expression of AhR, ERα, and CYP1A1 in MDA-MB-468, MDA-MB-231, Cal51, and MCF7 human breast cancer cells. (A). Whole cell lysates were collected from MDA-MB-468, MDA-MB-231, Cal51, and MCF7 human breast cancer cells. Western blotting indicates the relative protein expression levels of AhR and ERα in these cell lines. (B). Total RNA was collected from MDA-MB-468, MDA-MB-231, Cal51, and MCF7 human breast cancer cells and reverse transcribed. qPCR was performed for AHR and ESR1 (ERα) transcript, and the data is shown as mean relative mRNA level normalized to RPL13A, ± S.D of triplicate values. (C). Total mRNA was collected from MDA-MB-468, MDA-MB-231, Cal51, and MCF7 human breast cancer cells treated with 0.1% DMSO, 1 μM AF, or 1 μM BNF for 6 hours, and reverse transcribed. qPCR was used to determine the induction of the CYP1A1 gene (normalized to RPL13A), shown as ± S.D of triplicate values. [file 1471-2407-14-344-S2.docx]

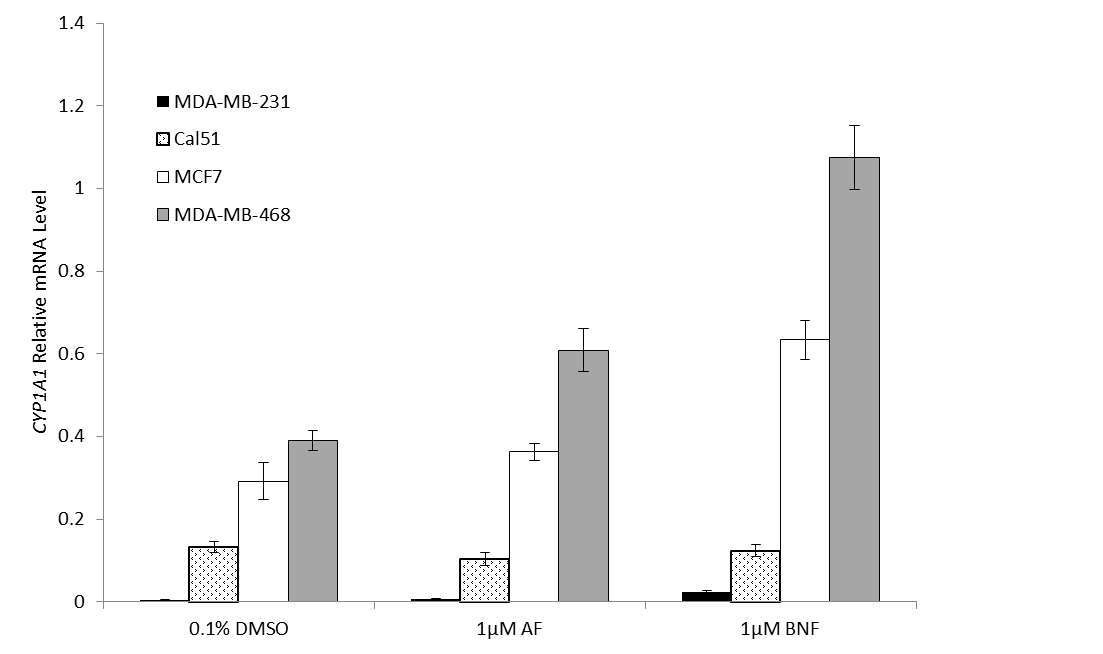

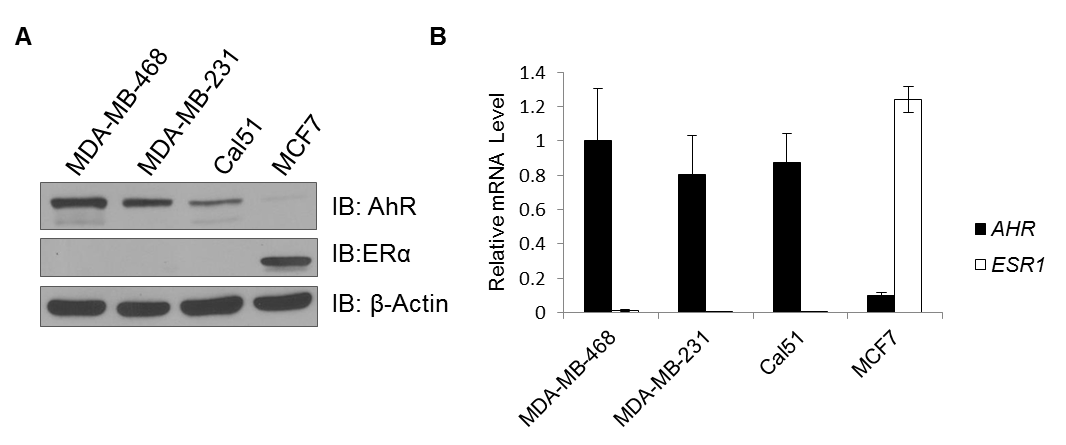


**Brinkman et al., Additional File 2 – Figure S1**

**C**

**Figure S1.** *Expression of AhR, ERα, and CYP1A1 in MDA-MB-468, MDA-MB-231, Cal51, and MCF7 human breast cancer cells.* **(A).**Whole cell lysates were collected from MDA-MB-468, MDA-MB-231, Cal51, and MCF7 human breast cancer cells. Western blotting indicates the relative protein expression levels of AhR and ERα in these cell lines. **(B).** Total RNA was collected from MDA-MB-468, MDA-MB-231, Cal51, and MCF7 human breast cancer cells and reverse transcribed. qPCR was performed for *AHR* and *ESR1* (ERα) transcript, and the data is shown as mean relative mRNA level normalized to *RPL13A*, ± S.D of triplicate values. **(C).** Total mRNA was collected from MDA-MB-468, MDA-MB-231, Cal51, and MCF7 human breast cancer cells treated with 0.1% DMSO, 1μM AF, or 1μM BNF for 6 hours, and reverse transcribed. qPCR was used to determine the induction of the *CYP1A1* gene (normalized to *RPL13A)*, shown as ± S.D of triplicate values.
