# Supplementary material for: Estrogen receptor α and aryl hydrocarbon receptor independent growth inhibitory effects of aminoflavone in breast cancer cells
Source: BMC Cancer. 2014 May 20;14:344. doi: 10.1186/1471-2407-14-344 (PMC4037283; doi:10.1186/1471-2407-14-344)
Supplement: Additional file 3: Figure S2 — AhR knockdown results in minimal alteration of SULT1A1 expression in MDA-MB-468shAhR and Cal51shAhR cells, while an efficient knockdown of SULT1A1 results in an increase in resistance to cytotoxicity mediated by AF. Total RNA was collected from MDA-MB-468shAhR (A) and Cal51shAhR (B) cells pretreated with 750 ng/mL Dox or vehicle for seven days to induce AhR knockdown, and subsequently treated with 0.1% DMSO, 5 μM BNF, or 5 μM AF for six hours. qPCR was performed for SULT1A1, and the data is shown as mean relative mRNA level normalized to RPL13A ± S.D. of triplicate values. SULT1A1 expression is minimally effected by AhR knockdown. (C). Total RNA was collected from parental MDA-MB-468 and Cal51 cells infected with lentivirus containing a scrambled shRNA or shRNA directed toward SULT1A1. qPCR was performed for SULT1A, and the data is shown as mean relataive mRNA level normalized to RPL13A ± S.D. of triplicate values. SULT1A1 knockdown appears to be efficient at the transcript level. 3-[4,5-dimethylthiazol-2-yl]-2,5 diphenyl tetrazolium bromide (MTT) assays were performed in (D) MDA-MB-468 cells harboring SULT1A1 shRNA and (E) Cal51 cells harboring SULT1A1 shRNA. Cells were plated in a 96-well format and treated with 0.1% DMSO or varying concentrations of AF for 48 hours prior to incubation with MTT. Knockdown of SULT1A1 results in enhanced resistance to AF-mediated cytotoxicity. **p < 0.01, *p < 0.05. [file 1471-2407-14-344-S3.docx]

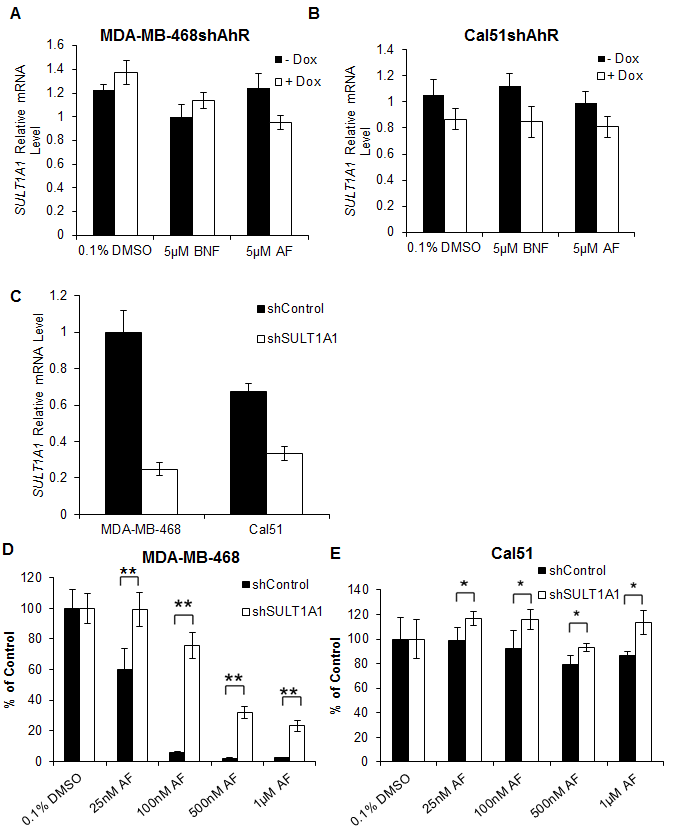


**Brinkman et al., Additional File 3 – Figure S2**

**Figure S2.** *AhR knockdown results in minimal alteration of SULT1A1 expression in MDA-MB-468shAhR and Cal51shAhR cells, while an efficient knockdown of SULT1A1 results in an increase in resistance to cytotoxicity mediated by AF.* Total RNA was collected from MDA-MB-468shAhR **(A)** and Cal51shAhR **(B)** cells treated witih 0.1% DMSO, 5μM BNF, or 5μM AF. qPCR was performed for *SULT1A1*, and the data is shown as mean relative mRNA level normalized to *RPL13A* ± S.D. of triplicate values. *SULT1A1* expression is minimally effected by AhR knockdown. **C.** Total RNA was collected from parental MDA-MB-468 and Cal51 cells infected with lentivirus containing a scrambled shRNA or shRNA directed toward SULT1A1. qPCR was performed for *SULT1A,* and the data is shown as mean relataive mRNA level normalized to *RPL13A* ± S.D. of triplicate values. *SULT1A1* knockdown appears to be efficient at the transcript level. 3-[4,5-dimethylthiazol-2-yl]-2,5 diphenyl tetrazolium bromide (MTT) assays were performed in **(D)** MDA-MB-468 cells harboring SULT1A1 shRNA and **(E)** Cal51 cells harboring SULT1A1 shRNA. Cells were plated in a 96-well format and treated with 0.1% DMSO or varying concentrations of AF for 48 hours prior to incubation with MTT. Knockdown of SULT1A1 results in enhanced resistance to AF-mediated cytotoxicity. **p<0.01, *p<0.05.
