# Supplementary material for: Estrogen receptor α and aryl hydrocarbon receptor independent growth inhibitory effects of aminoflavone in breast cancer cells
Source: BMC Cancer. 2014 May 20;14:344. doi: 10.1186/1471-2407-14-344 (PMC4037283; doi:10.1186/1471-2407-14-344)
Supplement: Additional file 4: Figure S3 — AhR is localized to both the cytoplasm and nucleus in Cal51 and MDA-MB-468 human breast cancer cells. Immunofluorescence for AhR was performed in Cal51 and MDA-MB-468, showing that AhR localizes to the cytoplasm, but also strongly in the nuclei of these cells. Images were acquired at 40×. [file 1471-2407-14-344-S4.docx]

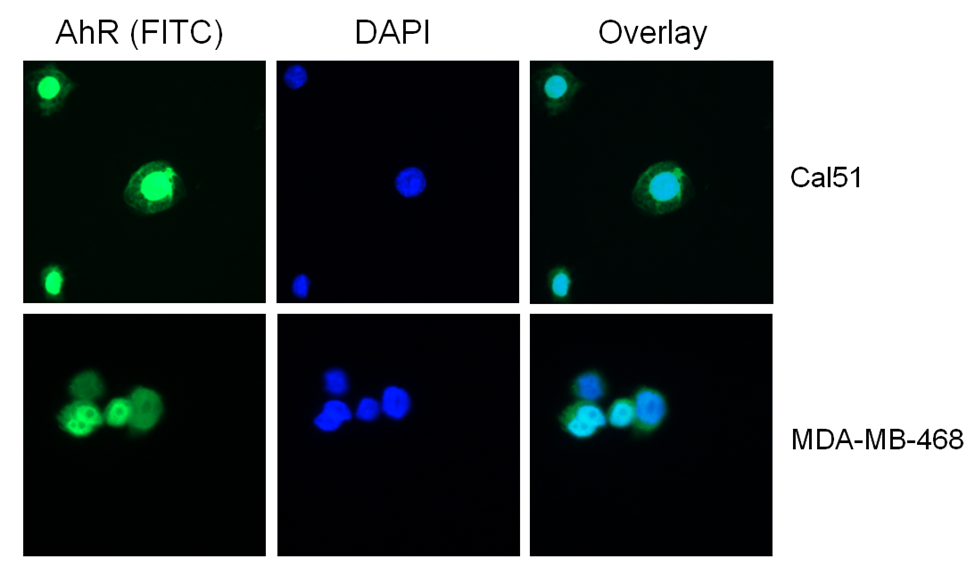


**Brinkman et al., Additional File 4 – Figure S3**

**Figure S3.** *AhR is localized to both the cytoplasm and nucleus in Cal51 and MDA-MB-468 human breast cancer cells.* Immunofluorescence for AhR was performed in Cal51 and MDA-MB-468, showing that AhR localizes to the cytoplasm, but also strongly in the nuclei of these cells. Images were acquired at 40X.
