# Supplementary material for: Estrogen receptor α and aryl hydrocarbon receptor independent growth inhibitory effects of aminoflavone in breast cancer cells
Source: BMC Cancer. 2014 May 20;14:344. doi: 10.1186/1471-2407-14-344 (PMC4037283; doi:10.1186/1471-2407-14-344)
Supplement: Additional file 5: Figure S4 — AF does not have significant effects on JNK activity in MDA-MB-468shAhR and Cal51shAhR cells. (A). Whole cell lysates were collected from MDA-MB-468shAhR pretreated with 750 ng/mL Dox and subsequently treated with 25nM AF, in the presence and absence of AhR knockdown by maintaining 750 ng/mL Dox or vehicle in the media. Western blotting shows that compared total c-Jun levels, phosphorylated c-Jun (p-c-Jun) does not appear to be affected by AF treatment. (B). Whole cell lysates were collected from Cal51shAhR pretreated with 750 ng/mL Dox and subsequently treated with 250 nM AF, in the presence and absence of AhR knockdown by maintaining 750 ng/mL Dox or vehicle in the media. Western blotting shows that compared total c-Jun levels, phosphorylated c-Jun (p-c-Jun) does not appear to be affected by AF treatment. We observe a decrease of total c-Jun protein at the 7 day time point. HSP90 was used as a loading control. [file 1471-2407-14-344-S5.docx]

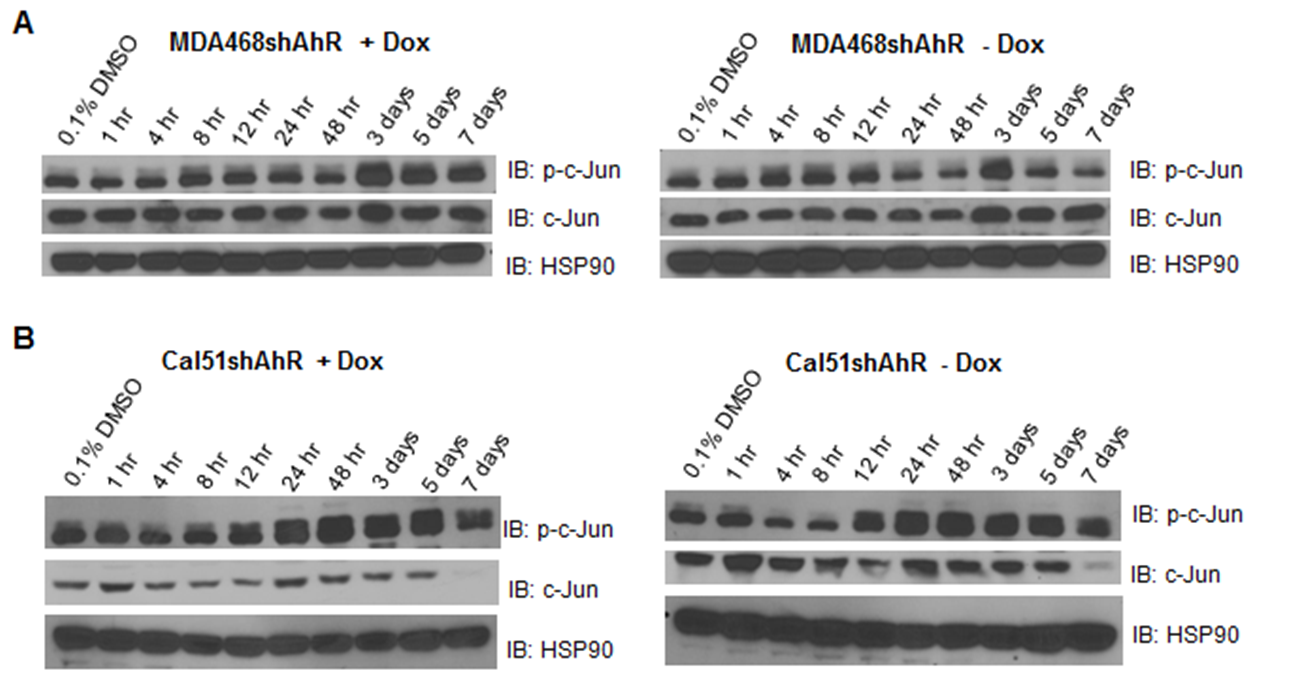


**Brinkman et al., Additional File 5 – Figure S4**

**Figure S4.** *AF does not have significant effects on JNK activity in MDA-MB-468shAhR and Cal51shAhR cells.* **(A).** Whole cell lysates were collected from MDA-MB-468shAhR pretreated with 750ng/mL Dox and subsequently treated with 25nM AF, in the presence and absence of AhR knockdown by maintaining 750ng/mL Dox or vehicle in the media. Western blotting shows that compared total c-Jun levels, phosphorylated c-Jun (p-c-Jun) does not appear to be affected by AF treatment. **(B).** Whole cell lysates were collected from Cal51shAhR pretreated with 750ng/mL Dox and subsequently treated with 250nM AF, in the presence and absence of AhR knockdown by maintaining 750ng/mL Dox or vehicle in the media. Western blotting shows that compared total c-Jun levels, phosphorylated c-Jun (p-c-Jun) does not appear to be affected by AF treatment. We observe a decrease of total c-Jun protein at the 7 day time point. HSP90 was used as a loading control.
