# Supplementary material for: Estrogen receptor α and aryl hydrocarbon receptor independent growth inhibitory effects of aminoflavone in breast cancer cells
Source: BMC Cancer. 2014 May 20;14:344. doi: 10.1186/1471-2407-14-344 (PMC4037283; doi:10.1186/1471-2407-14-344)
Supplement: Additional file 6: Figure S5 — Cyclin A2 increases in response to AF treatment in MDA-MB-468shAhR and Cal51shAhR cells. Whole cell lysates were collected from MDA-MB-468shAhR (A) and Cal51shAhR (B) pretreated with 750 ng/mL Dox and subsequently treated with 25nM AF or 250nM AF respectively, in the presence and absence of AhR knockdown by maintaining 750 ng/mL Dox or vehicle in the media. Western blotting shows that compared to control, AF causes an increase in Cyclin A2 protein in MDA-MB-468shAhR during the timecourse, both in the presence and absence of AhR knockdown, consistent with the observed S-phase cell cycle arrest. Cyclin A2 protein levels initially increase in Cal51shAhR, then decrease at the end of the timecourse, both in the presence and absence of AhR knockdown. This is consistent with the S-phase arrest observed in cell cycle analysis, with the 7 day (168 hour) time point having no statistically significant increase in percentage of S-phase cells. (C). Whole cell lysates were collected from MDA-MB-468shAhR pretreated with 750 ng/mL Dox and subsequently treated with 25nM AF, in the presence and absence of AhR knockdown by maintaining 750 ng/mL Dox or vehicle in the media. Western blotting shows that after 48 hours, 25nM AF causes PARP cleavage. [file 1471-2407-14-344-S6.docx]

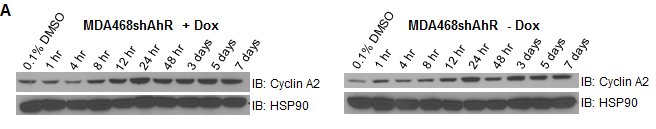


**Brinkman et al., Additional File 6 – Figure S5**


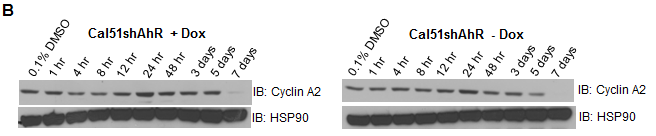


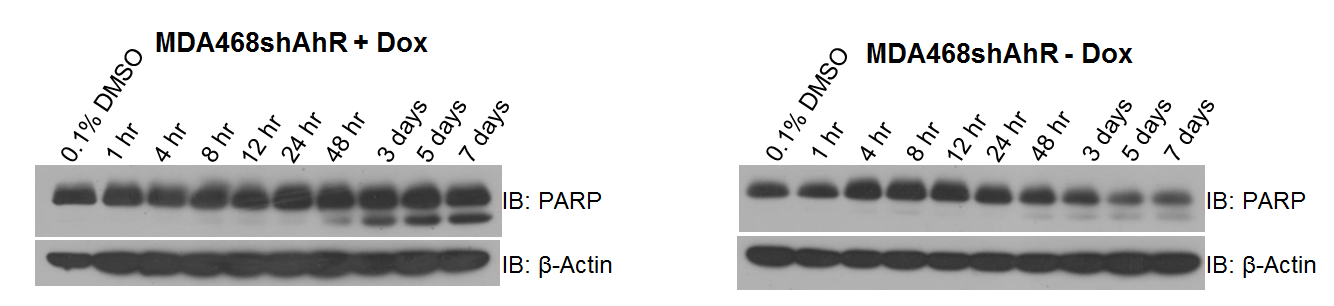


**C**

**Figure S5.** *Cyclin A2 increases in response to AF treatment in MDA-MB-468shAhR and Cal51shAhR cells.* Whole cell lysates were collected from MDA-MB-468shAhR **(A)** and Cal51shAhR **(B)** pretreated with 750ng/mL Dox and subsequently treated with 25nM AF or 250nM AF respectively, in the presence and absence of AhR knockdown by maintaining 750ng/mL Dox or vehicle in the media. Western blotting shows that compared to control, AF causes an increase in Cyclin A2 protein in MDA-MB-468shAhR during the timecourse, both in the presence and absence of AhR knockdown, consistent with the observed S-phase cell cycle arrest. Cyclin A2 protein levels initially increase in Cal51shAhR, then decrease at the end of the timecourse, both in the presence and absence of AhR knockdown. This is consistent with the S-phase arrest observed in cell cycle analysis, with the 7 day (168 hour) time point having no statistically significant increase in percentage of S-phase cells. **(C).** Whole cell lysates were collected from MDA-MB-468shAhR pretreated with 750ng/mL Dox and subsequently treated with 25nM AF, in the presence and absence of AhR knockdown by maintaining 750ng/mL Dox or vehicle in the media. Western blotting shows that after 48 hours, 25nM AF causes PARP cleavage.
