# Supplementary material for: Estrogen receptor α and aryl hydrocarbon receptor independent growth inhibitory effects of aminoflavone in breast cancer cells
Source: BMC Cancer. 2014 May 20;14:344. doi: 10.1186/1471-2407-14-344 (PMC4037283; doi:10.1186/1471-2407-14-344)
Supplement: Additional file 7: Figure S6 — The intensity of γ-H2AX staining is not proportional to AF dose in MDA-MB-468shAhR and Cal51shAhR cells. MDA-MB-468shAhR (A) and Cal51shAhR (B) were treated with a range of AF concentrations and then subjected to immunofluorescence staining for γ-H2AX. FITC (γ-H2AX) images were overlaid upon DAPI (nuclear), and at least thirty individual cells were assessed for intensity of γ-H2AX staining. We observed that γ-H2AX staining that remained constant regardless of AF dose. [file 1471-2407-14-344-S7.docx]

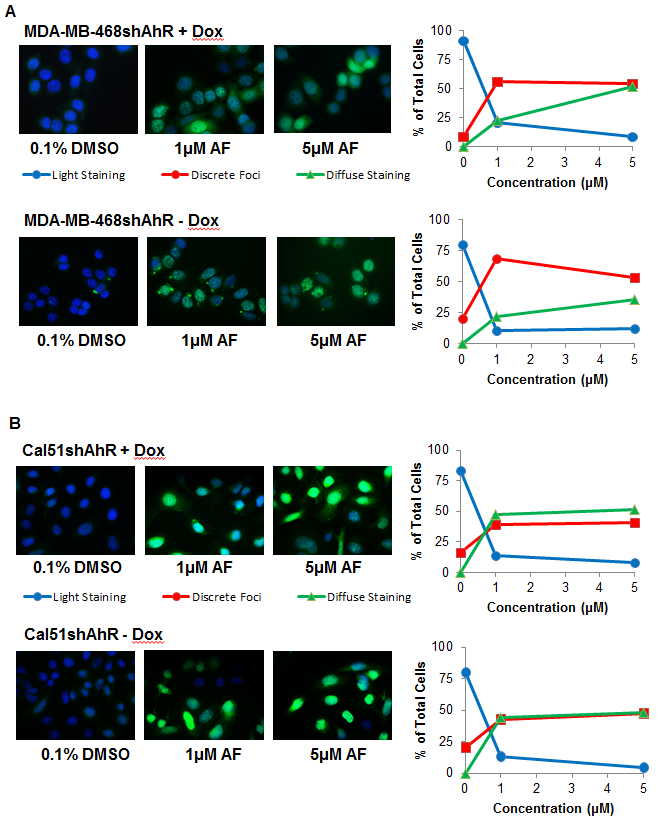


**Brinkman et al., Additional File 7 – Figure S6**

**Figure S6.** *The intensity of γ-H2AX staining is not proportional to AF dose in MDA-MB-468shAhR and Cal51shAhR cells.* MDA-MB-468shAhR **(A)** and Cal51shAhR **(B)** were treated with a range of AF concentrations and then subjected to immunofluorescence staining for γ-H2AX. FITC (γ-H2AX) images were overlaid upon DAPI (nuclear), and at least thirty individual cells were assessed for intensity of γ-H2AX staining. We observed that γ-H2AX staining that remained constant regardless of AF dose.
