# Supplementary material for: Estrogen receptor α and aryl hydrocarbon receptor independent growth inhibitory effects of aminoflavone in breast cancer cells
Source: BMC Cancer. 2014 May 20;14:344. doi: 10.1186/1471-2407-14-344 (PMC4037283; doi:10.1186/1471-2407-14-344)
Supplement: Additional file 8: Figure S7 — γ-H2AX staining intensity is not time dependent in MDA-MB-468shAhR and Cal51shAhR cells. MDA-MB-468shAhR (A) and Cal51shAhR (B) were treated with 25nM or 250nM AF respectively for six hours, then subjected to immunofluorescence staining for γ-H2AX. FITC (γ-H2AX) images were overlaid upon DAPI (nuclear), and at least thirty individual cells were assessed for intensity of γ-H2AX staining. We observed that γ-H2AX staining remained relatively constant over the timecourse. [file 1471-2407-14-344-S8.docx]

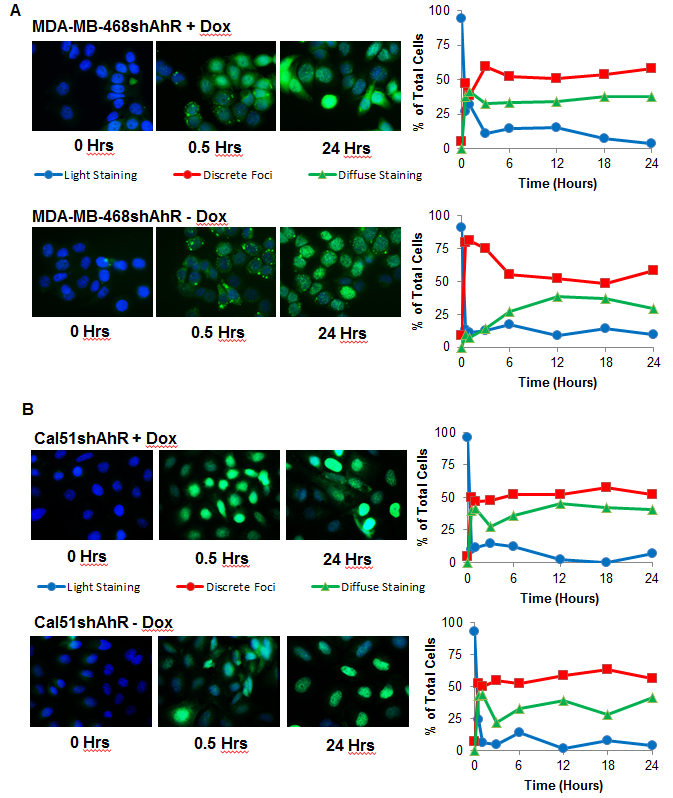


**Brinkman et al., Additional File 8 – Figure S7**

**Figure S7.** *γ-H2AX staining intensity is not time dependent in MDA-MB-468shAhR and Cal51shAhR cells.* MDA-MB-468shAhR **(A)** and Cal51shAhR **(B)** were treated with 25nM or 250nM AF respectively for six hours, then subjected to immunofluorescence staining for γ-H2AX. FITC (γ-H2AX) images were overlaid upon DAPI (nuclear), and at least thirty individual cells were assessed for intensity of γ-H2AX staining. We observed that γ-H2AX staining remained relatively constant over the timecourse.
