# Supplementary material for: Estrogen receptor α and aryl hydrocarbon receptor independent growth inhibitory effects of aminoflavone in breast cancer cells
Source: BMC Cancer. 2014 May 20;14:344. doi: 10.1186/1471-2407-14-344 (PMC4037283; doi:10.1186/1471-2407-14-344)
Supplement: Additional file 9: Figure S8 — γ-H2AX staining intensity is not reversed by the removal of AF for eight hours in MDA-MB-468shAhR and Cal51shAhR cells. MDA-MB-468shAhR (A) and Cal51shAhR (B) were treated with 25nM and 250nM AF respectively for six hours, then was replaced with untreated media for various lengths of time. Samples were subjected to immunofluorescence staining for γ-H2AX. FITC (γ-H2AX) images were overlaid upon DAPI (nuclear), and at least thirty individual cells were assessed for intensity of γ-H2AX staining. We observed that even after eight hours after AF removal, γ-H2AX staining persists, indicating that DNA damage mediated by AF may be irreversible in these cell lines. [file 1471-2407-14-344-S9.docx]

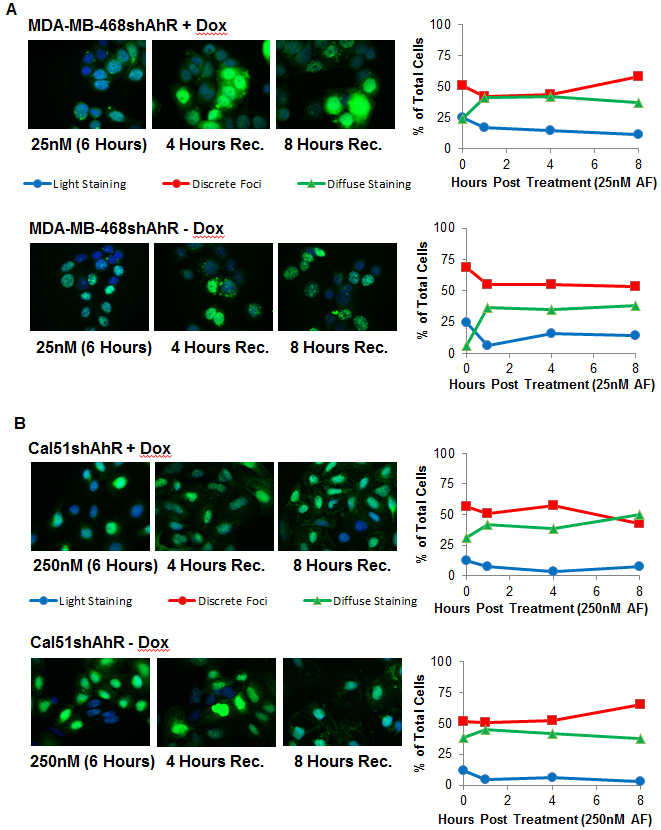


**Brinkman et al., Additional File 9 – Figure S8**

**Figure S8.** *γ-H2AX staining intensity is not reversed by the removal of AF for eight hours in MDA-MB-468shAhR and Cal51shAhR cells.* MDA-MB-468shAhR **(A)** and Cal51shAhR **(B)** were treated with 25nM and 250nM AF respectively for six hours, then was replaced with untreated media for various lengths of time. Samples were subjected to immunofluorescence staining for γ-H2AX. FITC (γ-H2AX) images were overlaid upon DAPI (nuclear), and at least thirty individual cells were assessed for intensity of γ-H2AX staining. We observed that even after eight hours after AF removal, γ-H2AX staining persists, indicating that DNA damage mediated by AF may be irreversible in these cell lines.
